# Trans-ancestral genome-wide association study of longitudinal pubertal height growth and shared heritability with adult health outcomes

**DOI:** 10.1186/s13059-023-03136-z

**Published:** 2024-01-16

**Authors:** Jonathan P. Bradfield, Rachel L. Kember, Anna Ulrich, Zhanna Balkhiyarova, Akram Alyass, Izzuddin M. Aris, Joshua A. Bell, K. Alaine Broadaway, Zhanghua Chen, Jin-Fang Chai, Neil M. Davies, Dietmar Fernandez-Orth, Mariona Bustamante, Ruby Fore, Amitavo Ganguli, Anni Heiskala, Jouke-Jan Hottenga, Carmen Íñiguez, Sayuko Kobes, Jaakko Leinonen, Estelle Lowry, Leo-Pekka Lyytikainen, Anubha Mahajan, Niina Pitkänen, Theresia M. Schnurr, Christian Theil Have, David P. Strachan, Elisabeth Thiering, Suzanne Vogelezang, Kaitlin H. Wade, Carol A. Wang, Andrew Wong, Louise Aas Holm, Alessandra Chesi, Catherine Choong, Miguel Cruz, Paul Elliott, Steve Franks, Christine Frithioff-Bøjsøe, W. James Gauderman, Joseph T. Glessner, Vicente Gilsanz, Kendra Griesman, Robert L. Hanson, Marika Kaakinen, Heidi Kalkwarf, Andrea Kelly, Joseph Kindler, Mika Kähönen, Carla Lanca, Joan Lappe, Nanette R. Lee, Shana McCormack, Frank D. Mentch, Jonathan A. Mitchell, Nina Mononen, Harri Niinikoski, Emily Oken, Katja Pahkala, Xueling Sim, Yik-Ying Teo, Leslie J. Baier, Toos van Beijsterveldt, Linda S. Adair, Dorret I. Boomsma, Eco de Geus, Mònica Guxens, Johan G. Eriksson, Janine F. Felix, Frank D. Gilliland, Penn Medicine Biobank, Torben Hansen, Rebecca Hardy, Marie-France Hivert, Jens-Christian Holm, Vincent W. V. Jaddoe, Marjo-Riitta Järvelin, Terho Lehtimäki, David A. Mackey, David Meyre, Karen L. Mohlke, Juha Mykkänen, Sharon Oberfield, Craig E. Pennell, John R. B. Perry, Olli Raitakari, Fernando Rivadeneira, Seang-Mei Saw, Sylvain Sebert, John A. Shepherd, Marie Standl, Thorkild I. A. Sørensen, Nicholas J. Timpson, Maties Torrent, Gonneke Willemsen, Elina Hypponen, Chris Power, The Early Growth Genetics Consortium, Mark I. McCarthy, Rachel M. Freathy, Elisabeth Widén, Hakon Hakonarson, Inga Prokopenko, Benjamin F. Voight, Babette S. Zemel, Struan F. A. Grant, Diana L. Cousminer

**Affiliations:** 1 Center for Applied Genomics, Children’s Hospital of Philadelphia, Philadelphia, PA 19104 USA; 2 Center for Spatial and Functional Genomics, Children’s Hospital of Philadelphia, Philadelphia, PA 19104 USA; 3 Department of Psychiatry, Perelman School of Medicine, University of Pennsylvania, Philadelphia, USA; 4 Department of Clinical & Experimental Medicine, University of Surrey, Guildford, UK; 5 Department of Metabolism, Digestion and Reproduction, Imperial College London, London, UK; 6 People-Centred Artificial Intelligence Institute, University of Surrey, Guildford, UK; 7 Department of Health Research Methods, Evidence, and Impact, McMaster University, Hamilton, Canada; 8 Division of Chronic Disease Research Across the Lifecourse, Department of Population Medicine, Harvard Medical School and Harvard Pilgrim Health Care Institute, Boston, MA 02215 USA; 9 MRC Integrative Epidemiology Unit at the University of Bristol, Bristol, UK; 10 Department of Genetics, University of North Carolina, Chapel Hill, NC USA; 11 Department of Population and Public Health Sciences, University of Southern California, Los Angeles, CA 90032 USA; 12 Saw Swee Hock School of Public Health, National University of Singapore and National University Health System, Singapore, Singapore; 13 Bristol Medical School, Population Health Sciences, University of Bristol, Bristol, UK; 14 K.G. Jebsen Center for Genetic Epidemiology, Department of Public Health and Nursing, NTNU, Norwegian University of Science and Technology, Trondheim, Norway; 15 ISGlobal, Barcelona, Spain; 16 Center for Life Course Health Research, University of Oulu, Oulu, Finland; 17 Department of Biological Psychology, Vrije Universiteit Amsterdam, Amsterdam, The Netherlands; 18 Department of Statistics and Computational Research, Universitat de València, Valencia, Spain; 19 CIBER Epidemiología y Salud Pública (CIBERESP), Madrid, Spain; 20 Epidemiology and Environmental Health Joint Research Unit, FISABIO-Universitat Jaume I-Universitat de València, Valencia, Spain; 21 Phoenix Epidemiology and Clinical Research Center, NIDDK, NIH, Bethesda, USA; 22 Institute for Molecular Medicine Finland, University of Helsinki, Helsinki, Finland; 23 Department of Clinical Physiology, Finnish Cardiovascular Research Center - Tampere, Faculty of Medicine and Health Technology, Tampere University, 33014 Tampere, Finland; 24 Department of Clinical Physiology, Tampere University Hospital, 33521 Tampere, Finland; 25 Wellcome Centre for Human Genetics, University of Oxford, Oxford, OX3 7BN UK; 26 Research Centre of Applied and Preventive Cardiovascular Medicine, University of Turku, Turku, Finland; 27 Centre for Population Health Research, University of Turku and Turku University Hospital, Turku, Finland; 28 Faculty of Health and Medical Sciences, Novo Nordisk Foundation Center for Basic Metabolic Research, University of Copenhagen, Copenhagen, Denmark; 29 Population Health Research Institute, St George’s, University of London, Cranmer Terrace, London, SW17 0RE UK; 30 Institute of Epidemiology, Helmholtz Zentrum München- German Research Center for Environmental Health, Neuherberg, Germany; 31 Division of Metabolic and Nutritional Medicine, Dr. Von Hauner Children’s Hospital, University of Munich Medical Center, Munich, Germany; 32 The Generation R Study Group, Erasmus MC, University Medical Center Rotterdam, Rotterdam, the Netherlands; 33 Department of Epidemiology, Erasmus MC, University Medical Center Rotterdam, Rotterdam, the Netherlands; 34 Department of Pediatrics, Erasmus MC, University Medical Center Rotterdam, Rotterdam, the Netherlands; 35 School of Medicine and Public Health, Faculty of Medicine and Health, University of Newcastle, Callaghan, NSW 2308 Australia; 36 Hunter Medical Research Institute, Newcastle, NSW 2305 Australia; 37 MRC Unit for Lifelong Health and Ageing at UCL, London, UK; 38 Department of Pediatrics, The Children’s Obesity Clinic, Copenhagen University Hospital Holbæk, Holbæk, Denmark; 39 Department of Pathology and Laboratory Medicine, Children’s Hospital of Philadelphia, Philadelphia, PA USA; 40 Faculty of Health and Medical Sciences, University of Western Australia, Perth, WA Australia; 41 Unidad de Investigación Médica en Bioquímica, Hospital de Especialidades, Centro Médico Nacional Siglo XXI, Instituto Mexicano del Seguro Social, Mexico City, Mexico; 42 MRC Centre for Environment and Health, School of Public Health, Faculty of Medicine, Imperial College London, St Mary’s Campus, Norfolk Place, London, W2 1PG UK; 43 Institute of Reproductive & Developmental Biology, Imperial College London, London, UK; 44 Center for Endocrinology, Diabetes & Metabolism, Children’s Hospital Los Angeles, Los Angeles, CA USA; 45 Haverford College, Haverford, PA USA; 46 Department of Pediatrics, Cincinnati Children’s Hospital, University of Cincinnati, Cincinnati, OH USA; 47 Department of Pediatrics, The University of Pennsylvania Perelman School of Medicine, Philadelphia, PA 19104 USA; 48 Division of Endocrinology & Diabetes, Children’s Hospital of Philadelphia, Philadelphia, PA 19104 USA; 49 College of Family and Consumer Sciences, University of Georgia, Athens, GA USA; 50 Singapore Eye Research Institute, Singapore National Eye Centre, Singapore, Singapore; 51 Department of Medicine and College of Nursing, Creighton University School of Medicine, Omaha, NB USA; 52 USC-Office of Population Studies Foundation, Inc, University of San Carlos, Cebu, Philippines; 53 Division of Gastroenterology, Hepatology and Nutrition, The Children’s Hospital of Philadelphia, Philadelphia, PA 19104 USA; 54 Department of Clinical Chemistry, Faculty of Medicine and Health Technology, Finnish Cardiovascular Research Center - Tampere, Tampere University, 33014 Tampere, Finland; 55 Department of Clinical Chemistry, Fimlab Laboratories, 33520 Tampere, Finland; 56 Department of Pediatrics and Adolescent Medicine, Turku University Hospital and University of Turku, Turku, Finland; 57 Department of Physiology, University of Turku, Turku, Finland; 58 Department of Nutrition, Harvard T.H Chan School of Public Health, Boston, MA 02115 USA; 59 Paavo Nurmi Centre, Unit for Health and Physical Activity, University of Turku, Turku, Finland; 60 Department of Nutrition, Gillings School of Global Public Health, University of North Carolina, Chapel Hill, NC USA; 61 Amsterdam Reproduction & Development (AR&D) Research Institute, Amsterdam, the Netherlands; 62 Universitat Pompeu Fabra (UPF), Barcelona, Spain; 63 Institute of Clinical Medicine Department of General Practice and Primary Health Care, University of Helsinki, Helsinki, Finland; 64 Folkhälsan Research Center, Helsinki, Finland; 65 Department of Obstetrics & Gynecology, Yong Loo Lin School of Medicine, National University of Singapore, Singapore, Singapore; 66 Cohort and Longitudinal Studies Enhancement Resources (CLOSER), UCL Institute of Education, London, UK; 67 The Faculty of Health and Medical Sciences, University of Copenhagen, Copenhagen, Denmark; 68 Department of Epidemiology and Biostatistics, School of Public Health, MRC-PHE Centre for Environment and Health, Imperial College London, London, W2 1PG UK; 69 Unit of Primary Health Care, Oulu University Hospital, OYS, Kajaanintie 50, 90220 Oulu, Finland; 70 Lions Eye Institute, Centre for Ophthalmology and Visual Science, Centre for Eye Research Australia, University of Western Australia, Perth, WA Australia; 71 Department of Pathology and Molecular Medicine, McMaster University, Hamilton, Canada; 72 Inserm UMR_S1256 Nutrition-Genetics-Environmental Risk Exposure, University of Lorraine, Nancy, France; 73 Department of Biochemistry-Molecular Biology-Nutrition, University Hospital Centre of Nancy, Nancy, France; 74 Division of Pediatric Endocrinology, Columbia University Medical Center, New York, NY USA; 75 Department of Maternity and Gynaecology, John Hunter Hospital, Newcastle, NSW 2305 Australia; 76 Metabolic Research Laboratory, School of Clinical Medicine, Wellcome-MRC Institute of Metabolic Science, University of Cambridge, Cambridge, CB2 0QQ UK; 77 MRC Epidemiology Unit, School of Clinical Medicine, Wellcome-MRC Institute of Metabolic Science, University of Cambridge, Cambridge, CB2 0QQ UK; 78 Department of Clinical Physiology and Nuclear Medicine, Turku University Hospital, Turku, Finland; 79 Department of Internal Medicine, Erasmus MC, University Medical Center Rotterdam, Rotterdam, the Netherlands; 80 Department of Epidemiology and Population Science, University of Hawaii Cancer Center, Honolulu, HI USA; 81 Department of Public Health, Faculty of Health and Medical Sciences, University of Copenhagen, Copenhagen, Denmark; 82 Fundació Institut d’Investigació Sanitària Illes Balears – IdISBa, Palma, Spain; 83 UCL Great Ormond Street Institute of Child Health, London, UK; 84 Australian Centre for Precision Health, Unit of Clinical and Health Sciences, University of South Australia, Adelaide, Australia; 85 South Australian Health and Medical Research Institute, Adelaide, Australia; 86 Department of Clinical and Biomedical Sciences, Faculty of Health and Life Sciences, University of Exeter, Exeter, EX2 5DW UK; 87 UMR 8199 - EGID, Institut Pasteur de Lille, CNRS, University of Lille, 59000 Lille, France; 88 Department of Genetics, University of Pennsylvania, Philadelphia, PA 19104 USA; 89 Department of Systems Pharmacology and Translational Therapeutics, Perelman School of Medicine, University of Pennsylvania, Philadelphia, PA 19104 USA; 90 Institute of Translational Medicine and Therapeutics, University of Pennsylvania, Philadelphia, PA 19104 USA; 91 Division of Human Genetics, Children’s Hospital of Philadelphia, Philadelphia, PA 19104 USA; 92 Currently Employed By GlaxoSmithKline, 1250 S Collegeville Rd, Collegeville, PA 19426 USA; 93 Current Address: Genentech, 1 DNA Way, San Francisco, CA 94080 USA

## Abstract

**Background:**

Pubertal growth patterns correlate with future health outcomes. However, the genetic mechanisms mediating growth trajectories remain largely unknown. Here, we modeled longitudinal height growth with Super-Imposition by Translation And Rotation (SITAR) growth curve analysis on ~ 56,000 trans-ancestry samples with repeated height measurements from age 5 years to adulthood. We performed genetic analysis on six phenotypes representing the magnitude, timing, and intensity of the pubertal growth spurt. To investigate the lifelong impact of genetic variants associated with pubertal growth trajectories, we performed genetic correlation analyses and phenome-wide association studies in the Penn Medicine BioBank and the UK Biobank.

**Results:**

Large-scale growth modeling enables an unprecedented view of adolescent growth across contemporary and 20th-century pediatric cohorts. We identify 26 genome-wide significant loci and leverage trans-ancestry data to perform fine-mapping. Our data reveals genetic relationships between pediatric height growth and health across the life course, with different growth trajectories correlated with different outcomes. For instance, a faster tempo of pubertal growth correlates with higher bone mineral density, HOMA-IR, fasting insulin, type 2 diabetes, and lung cancer, whereas being taller at early puberty, taller across puberty, and having quicker pubertal growth were associated with higher risk for atrial fibrillation.

**Conclusion:**

We report novel genetic associations with the tempo of pubertal growth and find that genetic determinants of growth are correlated with reproductive, glycemic, respiratory, and cardiac traits in adulthood. These results aid in identifying specific growth trajectories impacting lifelong health and show that there may not be a single “optimal” pubertal growth pattern.

**Supplementary Information:**

The online version contains supplementary material available at 10.1186/s13059-023-03136-z.

## Introduction

A distinct feature of human growth is the pubertal growth spurt, characterized by accelerated height growth. The characteristics of the growth spurt can differ substantially even among healthy children [1]. The timing of the onset of the pubertal growth spurt, the total amount of growth, and the duration of growth vary and are influenced by both genetic and environmental factors [2]. For instance, twin studies place the heritability of height growth during adolescence at about 75% [2, 3]. Furthermore, the secular trend of advancing pubertal timing (i.e., pubertal onset beginning at a younger age) in girls over the twentieth century has also been observed in boys by measuring peak height growth velocity (PHV) [3], the amount of height gained during the most rapid phase of height growth during puberty.

The timing and duration of the pubertal growth spurt affect the attainment of an individual’s final height, which is associated with, and a causal factor for, many adult health outcomes [4]. Thus, it is important to establish whether specific growth trajectory features impact these health risks. Indeed, epidemiological or observational studies have shown evidence that growth patterns during childhood are associated with health outcomes later in life, including adverse cardiovascular health [5], cancer [6, 7], bone outcomes such as lower bone mineral density in later life—a risk factor for osteoporosis [8, 9], type 2 diabetes [10–12], and respiratory health [13].

As noted above, variation in pubertal growth is highly heritable [2], but the specific genetic factors underlying pubertal growth trajectories remain largely unknown. In our previous study, we identified 10 genome-wide significant loci [14] using relatively simple phenotypes targeting the take-off phase of the growth spurt (the age in mid-childhood when growth velocity begins rising after falling since infancy), the total amount of pubertal growth, and the amount of late pubertal growth, which roughly marks the timing of PHV. The identified genetic signals influencing pubertal growth were also associated with pubertal timing, adiposity, and height growth potential. Half of the signals (5 out of 10) impacted height growth evenly across childhood and pubertal growth, while the other half were pubertal growth-specific, supporting the idea that some genetic factors contribute only during the growth spurt [2, 15–17]. We have also shown previously that independent signals (rs7759938 and rs314277) at one genetic locus (near *LIN28B*) can influence either postnatal growth from birth until adulthood or specifically pubertal growth [18], with the pubertal growth signal also being the strongest pubertal timing association in GWAS of age at menarche [19]. These signals have distinct associations with adult adiposity-related traits, with the puberty-associated signal also associated with adult height but not adiposity, whereas a second adult height-increasing signal also associated with increased weight and hip circumference [20].

To better capture the dynamic nature of the pubertal growth spurt, we expanded our previous work by utilizing longitudinal modeling of repeated height measurements across childhood and adolescence. Additionally, in contrast to our previous work, where we only included participants of European ancestry, we now include subjects from diverse ancestral backgrounds. Next, we uncovered genetic variants associated with longitudinal height growth and assessed the genetic associations between pubertal growth trajectories and later-life health traits.

## Results

### Modeled peak height velocity and age at peak height velocity across cohorts

**Fig. 1:**
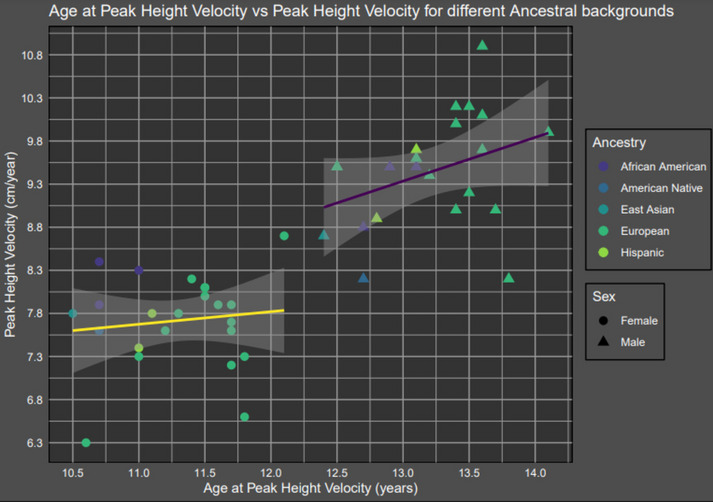
Mean peak height velocity (cm/year) vs. mean age at peak height velocity (years) for cohorts of diverse ancestral backgrounds. Yellow and purple lines represent a linear model fit to APHV ~ PHV × sex. The shaded bars represent the standard deviation of the linear regression. The samples included 19 cohorts of European ancestry, 3 African American cohorts, 2 East Asian cohorts, and 5 American Native or Hispanic cohorts

We performed Super-Imposition by Translation And Rotation (SITAR) growth curve analysis [21] in up to 56,659 samples (53.3% female; 41,468 European, 7852 African American, 2714 Asian, 2387 Native American, and 2238 Hispanic; Additional file 1: Table S1), including height measurements between age 5 and 20 years. SITAR estimates three random effects comparing each individual’s growth curve to the population mean, as well as predicted age at peak height velocity (APHV) and peak height velocity (PHV) for each overall population. Note that PHV in SITAR is the instantaneous peak velocity, which is greater than the peak annual velocity. We performed SITAR modeling separately in females and males separately in each contributing cohort. Overall, APHV across cohorts was consistent with the average ages reported previously (11.5 years in girls and 13.5 years in boys [22]), and non-European cohorts had earlier APHV than Europeans. There was a significant linear relationship between APHV (in years) and PHV (in cm/year) largely explained by sex (sex-adjusted *P* = 1.2 × 10^−15^; Fig. 1).

### Genome-wide association

**Fig. 2:**
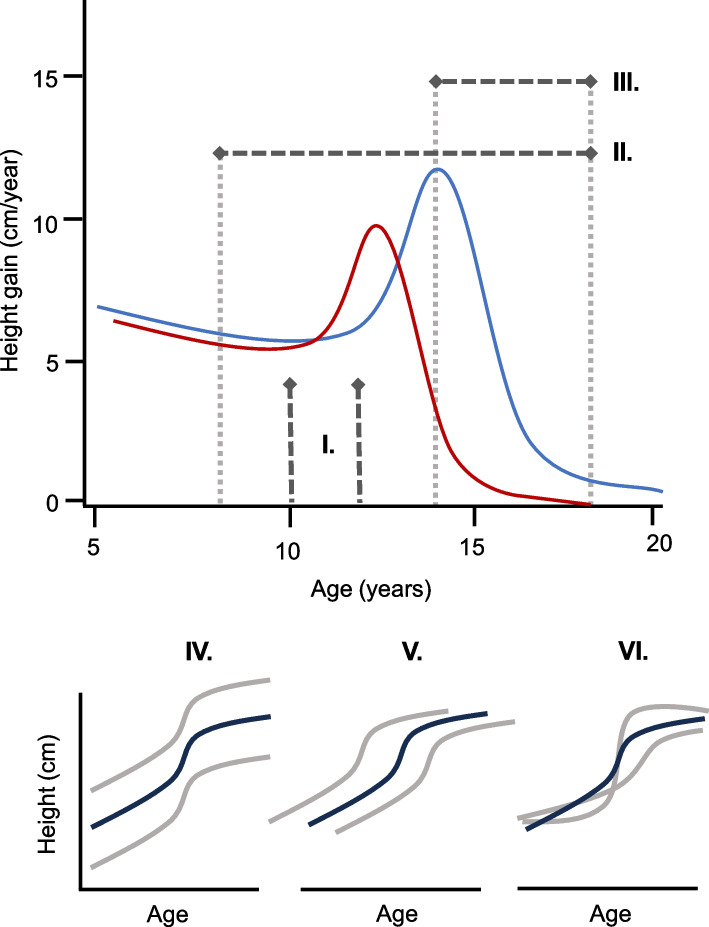
Six phenotypes assessed by GWAS. The top panel shows a typical growth curve for boys (blue) and girls (red), with age (years) on the *x*-axis and height gain (cm/year) on the *y*-axis. First, we included three simple height or height-difference phenotypes as previously assessed [14]: (I) The take-off phase of the pubertal growth spurt (height at age 10 in girls and 12 in boys); (II) total pubertal growth, between ages 8 and adult; and (III) late pubertal growth, between ages 14 and adult. Additionally, we included three phenotypes derived from SITAR longitudinal modeling: (IV) a-size; (V) b-timing; and (VI) c-intensity. The black line represents the mean population growth curve for a cohort (by sex). Each individual gets a random effect for the three parameters; for example, if a subject is taller than their peers (upper red line), they get a positive value for a-size, while a shorter individual (lower line in panel IV) gets a negative value. A subject who enters their growth spurt earlier than the mean (left line in panel V) gets a negative value for b-timing, while a subject growing later (right line in panel V) gets a positive value. Finally, a subject who grows faster than the population mean (steeper line in panel VI) receives a positive value for c-intensity, while a subject growing slower (shallower line in panel VI) gets a negative value for c-intensity

We performed a series of genome-wide association study (GWAS) analyses on six phenotypes: three simple height or height difference phenotypes, as previously assessed [14], and the three SITAR fixed effects (Fig. 2): (I) the take-off phase of the pubertal growth spurt (height at age 10 in girls and 12 in boys); (II) total pubertal growth, between ages 8 and adult (> 19 years); and (III) late pubertal growth, between ages 14 and adult (> 19 years). Additionally, we included three phenotypes derived from SITAR longitudinal modeling: (IV) *a-size*; (V) *b-timing*; and (VI) *c-intensity*. We ran ancestry-specific meta-analyses in the European, African American, Asian, and Native American/Hispanic ancestry groups, as well as a trans-ancestry meta-analysis followed by credible set analysis (Table 1, Additional file 1: Table S2). Twin studies suggest that height growth is most heritable during adolescence (up to 0.83 in boys and 0.76 in girls) [2, 23]; using Linkage Disequilibrium Score Regression (LDSC) [24], we calculated the SNP-heritability (*h*^*2*^_*SNP*_) of the six phenotypes and found five to have a genetic component: 10F/12 M = 0.32 (SE = 0.02); 8–adult = 0.22 (0.03); 14–adult = 0.21 (0.04); *a-size* = 0.15 (0.02); *c-intensity* = 0.13 (0.02); Additional file 1: Table S3).

**Table 1:** Trans-ancestry GWAS meta-analysis results (see Additional file 1)

Signal	Phenotype	Sex	MarkerName	Chromosome	Position (GRCh37)	EA	NEA	EAF	Nsample	Ncohort	beta	SE	*P*	Sample size	P_MR_Mega	P_ancestry_het	#SNPs_95_cred_set	Nearest gene	Functional candidate genes
1	I	M + F	rs67807996	1	149,995,265	A	G	0.31	35,014	39	0.067	0.009	**1.04E − 12**	34,534	**5.98E − 11**	1.44E − 01	1	OTUD7B	OTUD7B
2	I	M + F	rs823094	1	205,689,807	G	T	0.39	37,407	39	− 0.038	0.008	1.17E − 06	36,927	**2.30E − 08**	2.13E − 04	20	RAB7L1	NUCKS1
3	I	F	rs1346789	2	56,092,052	C	T	0.23	23,173	24	− 0.071	0.011	**1.03E − 09**	23,173	**4.28E − 08**	6.35E − 01	11	EFEMP1	EFEMP1, PNPT1
3	I	M + F	rs3791679	2	56,096,892	G	A	0.24	39,411	40	− 0.068	0.009	**7.52E − 15**	38,931	**1.02E − 12**	3.08E − 01	3	EFEMP1	EFEMP1, PNPT1
4	I	M + F	rs6763927	3	141,140,366	T	A	0.46	42,928	45	0.049	0.007	**4.01E − 12**	42,448	**4.96E − 10**	3.39E − 01	16	RASA2	ZBTB38
5	I	M + F	rs7623981	3	172,135,334	C	T	0.38	40,986	44	− 0.050	0.008	**4.46E − 11**	40,506	**2.67E − 09**	2.17E − 01	6	GHSR	GHSR
6	I	M + F	rs6817306	4	17,868,058	C	T	0.15	39,856	41	− 0.072	0.011	**1.93E − 11**	39,376	**1.91E − 10**	3.79E − 02	6	NCAPG	SLIT2
7	IV	F	rs1712374	4	82,154,710	C	T	0.38	21,200	19	0.082	0.012	**9.67E − 11**	21,200	**4.94E − 09**	5.25E − 01	27	PRKG2	PRKG2
7	IV	M + F	rs1443537	4	82,150,880	A	C	0.33	39,039	34	0.073	0.009	**1.31E − 14**	39,039	**4.17E − 12**	5.90E − 01	27	PRKG2	PRKG2
8	IV	F	rs6537315	4	145,724,712	A	G	0.36	21,645	20	− 0.066	0.012	1.21E − 07	21,645	**4.93E − 08**	9.26E − 03	11	HHIP	HHIP
8	I	M + F	rs2131354	4	145,599,908	A	G	0.47	40,541	43	0.044	0.007	**4.72E − 09**	40,061	**2.75E − 08**	4.76E − 02	40	HHIP	HHIP
9	I	M + F	rs409012	5	77,375,625	C	A	0.22	41,317	43	− 0.053	0.009	**1.60E − 09**	40,837	**3.98E − 08**	1.16E − 01	75	TBCA	AP3B1, OTP
10	IV	M + F	rs2814994	6	34,620,153	A	T	0.17	39,719	35	0.070	0.012	**1.65E − 09**	39,719	**2.79E − 08**	1.08E − 01	34	C6orf106 (ILRUN)	C6orf106 (ILRUN)
11	III	M + F	rs2181193	6	105,442,327	A	T	0.67	19,472	26	− 0.079	0.011	**7.81E − 13**	19,472	**9.59E − 11**	4.54E − 11	17	LIN28B	LIN28B
11	V	M + F	rs7766336	6	105,388,405	G	T	0.68	40,667	36	− 0.006	0.002	0.002158	40,667	**6.50E − 10**	1.01E − 08	10	LIN28B	LIN28B
11	VI	M + F	rs314263	6	105,392,745	T	C	0.69	39,893	36	0.007	0.001	7.25E − 07	39,893	**1.93E − 09**	4.41E − 05	38	LIN28B	LIN28B
12	I	F	rs1159619	6	126,801,144	A	C	0.46	21,466	22	0.063	0.010	**4.56E − 10**	21,466	**2.97E − 09**	1.22E − 01	129	CENPW	RSPO3
12	I	M + F	rs1361107	6	126,767,511	C	A	0.46	40,003	42	0.060	0.008	**3.05E − 15**	39,523	**1.64E − 13**	8.73E − 02	123	CENPW	CENPW
13	I	M + F	rs9496369	6	142,724,918	T	C	0.33	42,928	45	− 0.052	0.008	**2.36E − 11**	42,448	**2.00E − 09**	2.11E − 01	33	ADGRG6	ADGRG6
13	IV	M + F	rs7770386	6	142,775,657	T	C	0.35	39,833	35	− 0.052	0.009	**1.20E − 08**	39,833	**2.51E − 09**	1.65E − 03	14	ADGRG6	ADGRG6
14	I	F	rs2167733	8	78,110,734	A	G	0.24	23,618	25	0.073	0.011	**3.08E − 11**	23,618	**2.63E − 09**	9.30E − 01	39	PEX2	HNF4G
14	I	M + F	rs10103623	8	78,106,082	G	A	0.24	42,295	45	0.072	0.008	**2.87E − 18**	41,815	**2.30E − 15**	6.99E − 01	36	PEX2	PEX2
15	III	F	rs72829034	10	64,129,318	A	C	0.08	6660	10	− 0.005	0.033	8.79E − 01	6660	**1.30E − 09**	3.05E − 10	1	ZNF365	ARID5B
16	V	M + F	rs16911905	11	5,249,290	C	G	0.10	26,380	21	0.076	0.017	6.84E − 06	26,380	**1.32E − 09**	4.03E − 06	1	HBB	HBB
17	I	M + F	rs2724648	12	11,858,191	T	C	0.61	43,373	46	− 0.049	0.007	**1.58E − 11**	42,893	**6.62E − 09**	9.41E − 01	12	ETV6	ETV6
18	VI	M + F	rs2364232	12	93,994,827	C	A	0.25	42,873	40	0.008	0.001	**1.20E − 12**	42,873	**1.65E − 11**	7.54E − 02	5	SOCS2	SOCS2
19	I	M + F	rs28481863	12	123,902,361	A	G	0.77	41,317	43	− 0.054	0.009	**1.37E − 09**	40,837	**4.31E − 08**	1.58E − 01	155	KMT5A	KMT5A
19	IV	M + F	rs28406193	12	123,891,865	G	T	0.74	41,736	38	− 0.061	0.010	**7.43E − 10**	41,736	**1.14E − 08**	9.01E − 02	9	KMT5A	KMT5A
20	III	F	rs36103703	14	56,284,991	G	A	0.18	5929	11	− 0.035	0.025	1.56E − 01	5929	**3.82E − 09**	2.43E − 09	4	KTN1	KTN1
21	I	M + F	rs11634560	15	67,467,830	G	T	0.25	38,930	41	0.058	0.009	**7.58E − 11**	38,450	**1.98E − 08**	9.45E − 01	9	IQCH	SMAD3
22	I	M + F	rs10520573	15	84,591,575	G	A	0.57	41,431	45	0.049	0.007	**4.24E − 11**	40,951	**4.92E − 09**	4.69E − 01	69	UBE2Q2L	ADAMTSL3
23	I	M + F	rs71385734	16	2,160,503	G	T	0.20	37,390	38	− 0.058	0.010	**6.72E − 09**	36,910	**8.60E − 09**	8.80E − 03	8	PKD1	PKD1
24	I	F	rs7238093	18	20,728,158	A	T	0.20	21,683	23	− 0.081	0.012	**5.58E − 11**	21,683	**4.04E − 09**	8.60E − 01	15	CABLES1	CABLES1
24	IV	F	rs8094261	18	20,746,728	C	G	0.78	21,645	20	0.095	0.014	**5.46E − 11**	21,645	**4.89E − 09**	7.89E − 01	13	CABLES1	CABLES1
24	I	M + F	rs7244464	18	20,729,714	C	A	0.77	42,601	46	0.075	0.009	**2.16E − 18**	42,121	**3.70E − 15**	8.67E − 01	12	CABLES1	CABLES1
24	IV	M + F	rs7238093	18	20,728,158	T	A	0.80	42,740	40	0.081	0.011	**3.45E − 14**	42,740	**9.07E − 12**	5.14E − 01	13	CABLES1	CABLES1
25	I	M	rs143384	20	34,025,756	G	A	0.44	19,755	21	0.063	0.011	5.35E − 08	19,755	**1.22E − 08**	5.65E − 03	59	GDF5	GDF5
25	I	M + F	rs143384	20	34,025,756	G	A	0.45	41,711	44	0.060	0.008	**2.14E − 15**	41,231	**1.12E − 13**	7.34E − 02	1	GDF5	GDF5
25	IV	M + F	rs143384	20	34,025,756	G	A	0.49	41,850	38	0.063	0.009	**5.81E − 12**	41,850	**4.06E − 10**	2.40E − 01	67	GDF5	GDF5
26	I	M + F	rs1326022	20	54,844,025	G	A	0.69	37,469	39	− 0.053	0.008	**4.61E − 10**	36,989	**4.25E − 09**	4.83E − 02	19	MC3R	MC3R

Twenty-six loci achieved genome-wide significance (*P* < 5 × 10^−8^) in the trans-ancestral meta-analysis (Table 1). Four loci were significant for both height at 10F/12 M and *a-size*, and we noted that the *LIN28B* locus was associated with height difference age 14–adult, *b-timing*, and *c-intensity*. We then performed credible set analyses, and for 10 signals we reduced the 95% credible set to fewer than 10 SNPs (Additional file 1: Table S2). For three loci, the credible set was distilled down to a single SNP. Three example regional plots are shown in Additional file 2: Fig. S1.

We subsequently extracted all associations with other traits from previously published GWAS for these loci using PhenoScanner [25] (Additional file 1: Tables S4 and S5). All but three signals ((1) nearest to *ZNF365*, (2) nearest to the gene for hemoglobin beta, *HBB*, and (3) nearest to *LINC00520*) were genome-wide significantly associated with adult height, and all but four signals (the three above as well as at *LIN28B*) were associated with the UK Biobank phenotype “comparative height at age 10.” We then clustered the genome-wide significant loci based on their associations with the three SITAR parameters (Additional file 2: Fig. S2A) and found that most loci behaved similarly, again with the exception of the *LIN28B* and *HBB* loci. We observed that the *LIN28B* signal was associated with the timing and velocity of the pubertal growth spurt, as well as late pubertal growth, with a resulting impact on adult stature, but there was no effect on growth prior to puberty [14, 18].

The other outlying locus, near *HBB*, was associated with delayed *b-timing* (rs16911905, *P*_*Trans-Ancestry*_ = 1.32 × 10^−9^), but there was no evidence for association with childhood or adult stature based on previously published GWAS efforts. Investigating further, we found that the only cohort showing association was in the Children’s Hospital of Philadelphia African American cohort. The strongest association was at rs334 (beta (SE) = 0.342 (0.03), *P* = 5.80 × 10^−27^) (Additional file 2: Fig. S2B). rs334 is associated with sickle cell anemia, and children with sickle cell anemia have decreased pubertal growth velocity [26]. rs334 is only common in African populations, in which sickle cell anemia is most prevalent, so it did not pass quality control for the trans-ancestral analysis. When we conditioned for sickle cell anemia in the Children’s Hospital of Philadelphia African American cohort, we found that the signal was almost completely ablated (*P* = 0.03) (Additional file 2: Fig. S2C). Thus, this analysis likely picked up an association driven by children with sickle cell anemia in this cohort, who display reduced pubertal growth.

### Prioritized genes

Most GWAS signals are intergenic. Thus, we combined several approaches to identify the most likely effector gene for each locus. We looked for skeletal and body size aberrations in human Mendelian disease using OMIM and in mice knockout experiments (JAX, IMPC) and annotated the genes at each locus with Gene Ontology biological process terms. Altogether, we identified likely causal genes at 23 loci (Additional file 1: Table S6). These include several genes that result in severe ossification and/or skeletal abnormalities when dysregulated in mice, such as *PRKG2*, *ARID5B*, *SOCS2*, *SMAD3*, *PKD1*, and *GDF5*; of these, rare mutations in *PRKG2 *[27, 28], *SMAD3 *[29–31], and *GDF5 *[32–34] are also associated with rare Mendelian disorders in humans with skeletal phenotypes. GO-term-based pathway analysis revealed a significant enrichment of height at 10F/12 M with “regulation of chondrocyte differentiation” (Bonferroni-corrected *P* = 0.006).

### Genetic relationship between pubertal growth and anthropometric traits

Next, we explored the genetic correlation between pubertal growth traits and anthropometric and health-related outcomes in the European ancestry-specific results. The relationship between pubertal timing (e.g., its onset) and intensity (e.g., the speed of progression through puberty) remains controversial, with studies reporting discrepant findings [3, 4]; here, we found a negative genetic correlation between the SITAR-derived *b-timing* and *c-intensity* parameters (rg =  − 0.12; Additional file 1: Table S7; Additional file 2: Fig. S3), as well as a highly significant negative genetic correlation between *c-intensity* and age at menarche (rg =  − 0.52, *P* = 6.72 × 10^−23^), indicating that genetic determinants of later pubertal onset also favor a slower tempo of growth.

**Fig. 3:**
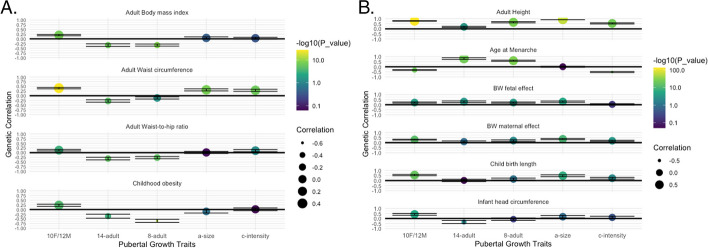
Genetic correlation (rg) between pubertal growth and anthropometric traits. **A** Correlation with adiposity traits. **B** Correlation with body size traits. 10F/12 M, height at age 10 years in girls and age 12 years in boys; 14–adult, height difference between age 14 years and adult; 8–adult, height difference between age 8 years and adult; a-size, SITAR-derived height across the growth trajectory; c-intensity, SITAR-derived tempo of the pubertal growth spurt

We then investigated anthropometric traits across the life course. In particular, the relationship between pubertal growth and BMI has been unclear. Here, *c-intensity* was not genetically correlated with childhood obesity or adult BMI (Fig. 3A), whereas taller height at 10/12 years was genetically correlated with higher childhood and adult BMI. Furthermore, more growth 8-to-adult and 14-to-adult were negatively genetically correlated with childhood and adult BMI. *C-intensity* was, however, positively genetically correlated with adult waist circumference, meaning that faster pubertal growth tempo correlated with larger adult waist circumference, as did taller *a-size* and height at 10F/12 M.

We observed a strong positive genetic correlation between *c-intensity* and adult height (rg = 0.54, *P* = 1.12 × 10^−23^), as well as between the other pubertal growth parameters and height across the life course (Fig. 3B) [23, 35]. Additionally, while *b-timing* would be expected to be correlated with the timing of puberty (not assessed here due to low heritability), we found that taller height at 10F/12 M, and higher *c-intensity* of the growth spurt, were genetically correlated with earlier age at menarche. Meanwhile, later age at menarche was genetically correlated with more growth from 8–adult and 14–adult, which is expected since adolescents who develop later have a longer period of growth.

### Relationship between the genetics of pubertal growth and later-life health outcomes

**Fig. 4:**
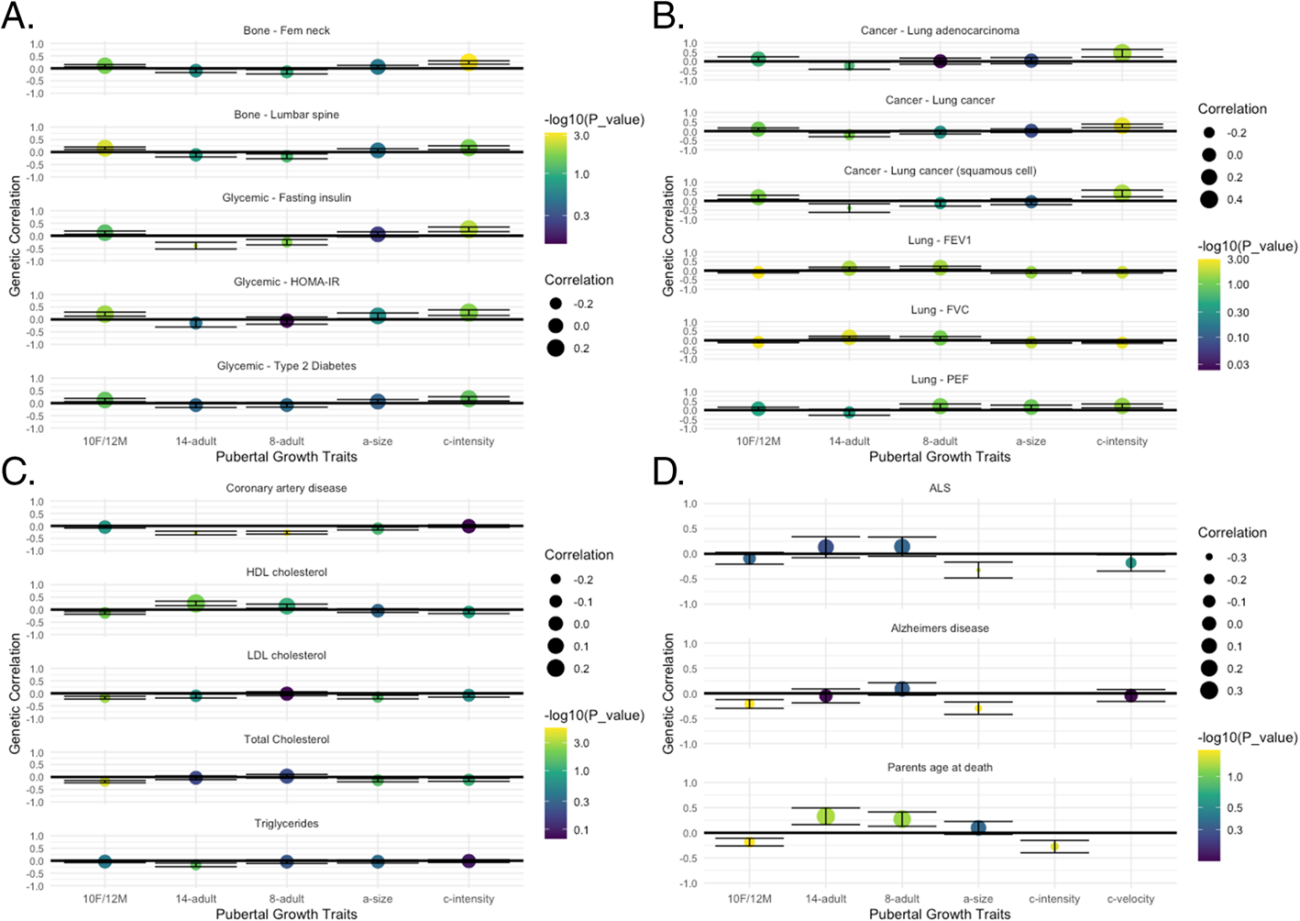
Genetic correlation (rg) of pubertal growth with adult health outcomes**. A** Bone and glycemic outcomes, including femoral neck and lumbar spine bone mineral density and three glycemic traits. **B** Lung cancer and lung function outcomes. **C** Cardiac and lipid outcomes. **D** Neuropsychiatric outcomes and overall wellbeing (parents’ age at death). LDL, low-density lipoprotein; HDL, high-density lipoprotein; HOMA-IR, homeostatic model assessment for insulin resistance; PEF, peak expiratory flow; FVC, forced vital capacity; FEV1, forced exhalation volume in 1 s; ALS, Amyotrophic lateral sclerosis. 10F/12 M, height at age 10 years in girls and age 12 years in boys; 14–adult, height difference between age 14 years and adult; 8–adult, height difference between age 8 years and adult; a-size, SITAR-derived height across the growth trajectory; c-intensity, SITAR-derived tempo of the pubertal growth spurt

Variation in growth during puberty is known to be associated with later-life health outcomes [5, 6, 8–13]. Here, analysis of both the genome-wide significant signals and genome-wide genetic correlations pointed towards pleiotropy between pubertal growth and health outcomes. With LDSC, which compares genome-wide association data across the genome between pairs of traits, we observed genetic correlations with bone mineral density, cardiovascular traits such as atrial fibrillation and coronary artery disease, glycemic traits, lung function and lung cancer, neurological, psychiatric, and intelligence traits, and overall well-being (Fig. 4).

With evidence of a genetic relationship between pubertal growth and later health outcomes, we next generated polygenic risk scores (PRS) from sets of genetic variants (see the “Methods” section, Additional file 1: Table S8) and performed phenome-wide association (PheWAS) scans on individual-level data in the Penn Medicine Biobank (PMBB) and the UK Biobank. In the PMBB, no phenotypes passed Bonferroni multiple testing correction. However, 140 traits showed a nominal association (*P* < 0.05, Additional file 1: Table S9). We performed validation analyses in the UK Biobank, selecting 37 traits that demonstrated either significant LDSC-based genetic correlations or nominally significant associations in the PMBB. Although the proportions of phenotypic variance explained by the PRS were small, three phenotypes remained significantly associated with one or more PRS after multiple test correction in the UK Biobank: atrial fibrillation, type 2 diabetes, and adult BMI (Table 2, Additional file 1: Table S10). Notably, additional cardiac traits were nominally associated with the PRS.

**Table 2:** Polygenic risk score results in the UK Biobank

Phenotype	PRS *R*^2 a^	Full model R^2 b^	PRS P	Number SNPs in PRS
**I (10F/12 M)**				
Atrial fibrillation diagnosis	0.000215	0.030	3E − 25	4133
BMI	8E − 05	0.017	3E − 10	4133
Extreme BMI	2E − 05	0.003	0.0006	4133
T2D all	2E − 05	0.032	0.001	4133
**IV (a-size)**				
Atrial fibrillation diagnosis	3E − 05	0.030	0.0003	383
**V (b-timing)**				
BMI	2E − 05	0.017	0.0005	350
**VI (c-intensity)**				
BMI	6E − 05	0.017	5E − 08	457
Atrial fibrillation diagnosis	2E − 05	0.030	0.0006	457

^a^Proportion of variance explained by the polygenic risk score (PRS)

^b^Proportion of variance explained by the full model including covariates (array, sex, age, PC1, PC2, PC3, PC4, PC5, PC6) and PRS

## Discussion

In this study, we assessed children and adolescents of multiple ancestries with repeated height measurements and genetic data to gain a better understanding of how pubertal height growth relates to health across the life course. First, we performed longitudinal modeling by sex and ancestry with SITAR, a validated method for producing more precise and less biased estimates of APHV and PHV [36, 37]. Previously, SITAR has been used to estimate these parameters in individual cohorts [38, 39]. Here, we compared these estimates across cohort studies of different ancestral backgrounds collected over a range of years, from the 1930s to the present day. Studies of growth in healthy children have reported a mean APHV at around 11.5 years in girls and 13.5 years in boys [22]. Overall, the SITAR-modeled APHV values are consistent, with variation in mean age by ancestral background (non-European earlier than European), sex (females earlier than males), and the era of cohort collection (contemporary cohorts earlier than those collected decades ago).

The relationship between pubertal timing and intensity is controversial, with studies reaching various conclusions. For example, Marceau et al. [40] found a relationship between timing and intensity for boys, but not girls, whereas German et al. [41] observed a negative correlation between pubertal onset and progression in girls. Our genetic correlation results support the latter, with a strong negative correlation between age at menarche and *c-intensity*. However, in Fig. 1, we see a weaker positive correlation between APHV and PHV. Additionally, higher childhood BMI is an established risk factor for earlier pubertal onset [42], but its relationship with the intensity of puberty is less clear than with pubertal timing. German et al. found that higher childhood BMI did not correlate with intensity, but instead with earlier pubertal timing. Our results corroborate these findings.

For final adult height, our findings diverge from previous studies that have found that the timing or intensity of pubertal development is unrelated to final stature [43, 44]. These previous findings could be due to small sample sizes. Here, with longitudinal data on > 50,000 adolescents, we show that genetic determinants of pubertal growth not only impact final stature, but measures of body size from birth to adulthood.

Utilizing genetic data, we performed multi-ancestry GWAS of pubertal growth. While the loci we identified are mostly known height loci, we report an association with the tempo (*c-intensity*) of pubertal growth at *LIN28B*, adding to its known associations with pubertal timing and childhood growth trajectories [14, 18].

While epidemiological studies have observed relationships between puberty and adult health outcomes, our large sample size and genetic data allowed us to explore genetic relationships using genetic correlation analyses and PRS in two large biobanks (PMBB and UKBiobank). Our results support genetic relationships between pubertal growth and a range of adult-health traits. For instance, previous studies have identified later puberty and slower growth velocity as risk factors for later-life decline in bone density [9] and increased fracture risk [8]. Here, we observed a similar genetic relationship between slower *c-intensity* of growth and lower adult bone mineral density. For glycemic traits, previous studies showed that accelerated childhood growth associated with increased type 2 diabetes risk [10, 11], which corroborates with our observation of a positive correlation between *c-intensity* and type 2 diabetes.

The most consistent relationship was that of pubertal growth with cardiac traits. Shorter adult stature and less growth from age 7 to 13 years are risk factors for coronary heart disease [45], and we observed a similar genetic correlation between diminished growth 8–adult and 14–adult and coronary heart disease. To the contrary, greater pubertal growth was positively correlated with atrial fibrillation, flutter, and dysrhythmias. Both birthweight [46] and adult height [4] are known causal risk factors for atrial fibrillation, and several studies show relationships between increasing height and atrial fibrillation incidence [47–50]. These findings support the idea that body height has a lifelong impact on atrial fibrillation risk.

Our study does have some limitations. In our data, the *b-timing* parameter was not heritable, although the timing of puberty is well-established as a heritable trait; thus, we were unable to perform adult heath genetic correlation analyses with this trait. This could be due to our use of age rather than log(age) in the SITAR modeling, but we do detect well-established loci associated with pubertal timing despite the low heritability; in the future, studies running SITAR modeling with log(age) may provide more accurate estimates for b-timing. Furthermore, we did not study gene by environment interactions, which could be important given that pubertal timing is strongly affected by environmental factors; this analysis was outside the scope of the current study Additionally, not all cohorts were able to collect height data annually or bi-annually; thus, estimation of APHV in these cohorts may not be as precise. However, annual height measurements have been shown to estimate APHV as accurately as more frequent measurements [51]. A fuller understanding of the complex relationship between the genetic variation that impacts pubertal timing and adult health outcomes would be achieved by adding other factors (e.g., cumulative estimates of environmental risk factor exposure) to statistical models, which could be pursued in future investigations. Finally, the sample sizes for some of the ancestry groups remain small. In the future, we hope additional non-European datasets with longitudinal height measurements will become available.

## Conclusions

Here, we present the first trans-ancestry genetic study of childhood and adolescent growth. Large-scale growth modeling data allowed an unprecedented view of APV and PHV across contemporary and 20th-century pediatric cohorts. Our data supports genetic relationships between pediatric height growth and health across the life course, with different growth trajectories correlated with different outcomes. Being taller at early puberty associated with less growth across puberty; conversely, measures of growth from birth until adulthood were genetically correlated. Meanwhile, being shorter at age 10/12 for girls/boys correlated with a slower intensity of pubertal growth and later age at menarche. In terms of adult health outcomes, a faster intensity of pubertal growth correlated with higher BMD, HOMA-IR, fasting insulin, and T2D, and lung cancer, and being taller at early puberty and taller across puberty as well as having quicker pubertal growth tempo were associated with higher risk for atrial fibrillation. These results show that there may not be a single “optimal” pubertal growth pattern and highlight the importance of adolescent growth for later life health.

## Methods

### Contributing studies and phenotypes

Twenty-two cohorts contributed to this study (Additional file 1: Table S1), some with more than one ancestral background. These included up to 41,468 samples of European ancestry (EUR) in 19 cohorts, 7852 African American samples (AFR) in 3 cohorts, 2714 East Asian (EAS) in 2 cohorts, and 4625 American Native or Hispanic (AMR) in 5 cohorts. In total, we included data from 26,478 boys and 30,181 girls aged 5–18 years. Height was measured using standard practices [6]. Details on the range, average, and median number of measurements per cohort is provided in Supplementary Table 1.

Each cohort individually modeled height growth using SITAR following a standard protocol.

For cohorts with sparse height measurements, modeling was performed together with the BMDCS or ALSPAC cohorts as a reference (see Additional file 1: Table S1). The SITAR random effects *a-size*, *b-timing*, and *c-intensity* were used as input phenotypes for GWAS, in addition to three simple height or height difference phenotypes: standardized height at age 10 in girls or age 12 in boys, standardized height difference between age 8 and adult (> 19 years), and standardized height difference between age 14 and adult (> 19 years) (these phenotypes are previously described [14]).

### Genome-wide association and meta-analysis

Each cohort individually performed genotyping and GWAS. Genotyping was performed on Illumina or Affymetrix genotyping arrays with centrally recommended post-genotyping quality control (QC). This included a sample call rate < 0.95, autosomal heterozygosity rate > 3 standard deviation from mean, SNP call rate < 0.98, mismatching reported and genotype-based sex, Hardy–Weinberg equilibrium (HWE) *p* < 1 × 10^−6^, and MAF < 0.01. We also excluded SNPs with high duplicate discordance rates and monomorphic SNPs. When possible, imputation was performed against the Haplotype Reference Consortium v.1.0 or 1.1 [52]; other population-specific panels were used for specific studies (SISU v2 for HBCS; Pima Indian-specific panel for the Southwest American Indians; 1000 Genomes for SCORM, NSHD, CLHNS). Following imputation, it was recommended to only remove monomorphic SNPs and to not filter on any other criteria as pre-meta-analysis QC would be performed centrally. Next, each cohort performed GWAS using cohort-specific covariates (Additional file 1: Table S1). Post-imputation QC was then performed centrally for all studies and included filters for MAF < 0.05, HWE, and imputation quality (excluding INFO < 0.4). EasyQC [53] was used to perform cohort-specific QC and meta-level QC. Ancestry-specific meta-analysis was then performed with GWAMA using 2 rounds of genomic control to correct for population.

### Trans-ancestral meta-analysis

Meta-Regression of Multi-Ethnic Genetic Association (MR-Mega) [54] was used to perform meta-analysis on all cohorts to account for differences in cohort ancestry. A female only, male only, and all sex combined meta-analysis was performed for each of the six phenotypes. Post meta-analysis SNPs were filtered out if they had MAF < 0.05 or were present in less than 50% of the cohorts. Manhattan plots and QQ plots are shown in Additional file 2: Fig. S4 and S5, respectively. Lambda values for each GWAS are given in Additional file 1: Table S11.

### Credible set analysis

The script credible_set_analysis.py was used to calculate the 95% credible sets for every genome-wide significant locus. The sum of the posterior probabilities was calculated from a sorted list of the most significant Bayes’ factors until the cumulative sum was equal to or greater than 0.95. This set of SNPs was then considered the 95% credible set.

### Functional annotation (FUMA, OpenTargets, Phenoscanner)

Trans-ancestry summary statistics were uploaded to Functional Mapping and Annotation of Genome-Wide Association Studies (FUMA GWAS; https://fuma.ctglab.nl/) to provide detailed annotation of the GWAS results [55]. PhenoScanner V2 (http://www.phenoscanner.medschl.cam.ac.uk/) was used to look up significant sentinel signals and their proxies (*r*^2^ = 0.8) to gather all statistically significant GWAS associations with other traits and diseases [25, 56]. OpenTargets (https://www.genetics.opentargets.org/) was further used to gather functional information on sentinel SNPs and target genes. Additionally, functional information on potential effector genes was gathered from the Online Mendelian Inheritance in Man (OMIM) database (https://www.ncbi.nlm.nih.gov/omim), the International Mouse Phenotyping Consortium (IMPC) database (https://www.mousephenotype.org/), and the Mouse Genome Informatics database (http://www.informatics.jax.org/).

### Genetic correlations

LD Score Regression [24], either on http://ldsc.broadinstitute.org/ or using python scripts downloaded from GitHub (https://github.com/bulik/ldsc), was used to perform genetic correlation analyses between the pubertal growth phenotypes (European meta-analyses) and outcome traits.

### PMBB PheWAS

The Penn Medicine BioBank (PMBB) recruits participants through the University of Pennsylvania Health system. At the time of medical appointment, participants give informed consent to access their electronic health records and donate a blood sample for DNA analysis. Genotyping, imputation, and phenotyping of this sample have been described in detail previously [57]. For the analysis presented here, we used genotype and phenotype data for 10,182 European ancestry individuals.

As the primary phenotypes for polygenic risk scores are not readily available in the PMBB cohort due to it being an adult sample, we identified the best performing PRS in a leave-one-out analysis, and then used that PRS in PMBB. First, we repeated the meta-analysis in European ancestry cohorts only, leaving out the CHOP Center for Applied Genomics (CAG) cohort. SNPs were then restricted to a common set present in all three datasets: the meta-analysis, CAG, and PMBB: 1 = 5,070,548, 2 = 5,065,379, 3 = 5,035,856, 4 = 5,035,192, 5 = 5,034,759, 6 = 5,035,034. Multiple candidate polygenic risk scores at nine p-value thresholds (× 10^−6^, 1 × 10^−5^, 1 × 10^−4^, 1 × 10^−3^, 0.01, 0.05, 0.1, 0.5, 1) were calculated in European individuals from the CAG cohort using PRSice, with 2,500 randomly selected European ancestry samples from CAG used to estimate LD (r^2^ > 0.1). Each candidate PRS for each phenotype was then tested against the corresponding phenotype in the CAG cohort in order to find the optimal p-value threshold. The SNPs included in the candidate PRS that produced the strongest association for each phenotype were then used to create the PRS in the PMBB cohort (Additional file 1: Table S8).

In the PMBB, PRS were created in 10,182 European ancestry individuals in PLINK 1.9 using the SNPs from the best performing PRS in CAG, and the weights derived from the leave-one-out meta-analysis. PRS were standardized with mean = 0 and SD = 1. A PheWAS was performed using logistic regression models with the PRS as the independent variable, phecodes as the dependent variable, and age, sex, and the first 10 principal components (PCs) as covariates. Phecodes with > 100 cases were tested (*N* = 512).

### UKBB PheWAS

We used the UK Biobank (UKBB) cohort with imputed genotype and electronic health record data to replicate nominally significant PMBB PheWAS findings and LDSC genetic correlations. Details on genotyping, imputation, and phenotyping in UKBB have been published elsewhere [58]. The best performing PRS—as described above in the CAG cohort—was constructed and simultaneously tested for their associations with 57 binary (*N* = 421,679) and 26 continuous phenotypes (*N*_max_ = 486,248) using PRSice-2 [59]. Disease status was defined using ICD10 codes from hospital admission data released in March 2020 and only outcomes with > 100 cases were included. Binary phenotypes were tested in logistic regression while continuous phenotypes in linear regression models. Covariates included age, sex, genotyping array, and the first six genetic principal components in all models.

### Supplementary Information

**Additional file 1:  Table 1 and tables S1-S11.**

**Additional file 2.** Figures S1-S5, Cohort funding and acknowledgments.

**Additional file 3.** Peer review history

## Data Availability

The SITAR code used in this study is available at https://github.com/DCousminer/EGG_SITAR and 10.5281/zenodo.10214403 Credible set analysis was performed using https://github.com/edm1/Credible-set-analysis/blob/master/credible_set_analysis.py. GWAS summary stats will be deposited in the GWAS catalog database at publication.
