# Author Correction: Trans-ancestral genome-wide association study of longitudinal pubertal height growth and shared heritability with adult health outcomes

**DOI:** 10.1186/s13059-024-03276-w

**Published:** 2024-05-21

**Authors:** Jonathan P. Bradfeld, Rachel L. Kember, Anna Ulrich, Zhanna Balkhiyarova, Akram Alyass, Izzuddin M. Aris, Joshua A. Bell, K. Alaine Broadaway, Zhanghua Chen, Jin-Fang Chai, Neil M. Davies, Dietmar Fernandez-Orth, Mariona Bustamante, Ruby Fore, Amitavo Ganguli, Anni Heiskala, Jouke-Jan Hottenga, Carmen Íñiguez, Sayuko Kobes, Jaakko Leinonen, Estelle Lowry, Leo-Pekka Lyytikainen, Anubha Mahajan, Niina Pitkänen, Theresia M. Schnurr, Christian Theil Have, David P. Strachan, Elisabeth Thiering, Suzanne Vogelezang, Kaitlin H. Wade, Carol A. Wang, Andrew Wong, Louise Aas Holm, Alessandra Chesi, Catherine Choong, Miguel Cruz, Paul Elliott, Steve Franks, Christine Frithiof-Bøjsøe, W. James Gauderman, Joseph T. Glessner, Vicente Gilsanz, Kendra Griesman, Robert L. Hanson, Marika Kaakinen, Heidi Kalkwarf, Andrea Kelly, Joseph Kindler, Mika Kähönen, Carla Lanca, Joan Lappe, Nanette R. Lee, Shana McCormack, Frank D. Mentch, Jonathan A. Mitchell, Nina Mononen, Harri Niinikoski, Emily Oken, Katja Pahkala, Xueling Sim, Yik-Ying Teo, Leslie J. Baier, Toos van Beijsterveldt, Linda S. Adair, Dorret I. Boomsma, Eco de Geus, Mònica Guxens, Johan G. Eriksson, Janine F. Felix, Frank D. Gilliland, Penn Medicine Biobank, Torben Hansen, Rebecca Hardy, Marie-France Hivert, Jens-Christian Holm, Vincent W. V. Jaddoe, Marjo-Riitta Järvelin, Terho Lehtimäki, David A. Mackey, David Meyre, Karen L. Mohlke, Juha Mykkänen, Sharon Oberfeld, Craig E. Pennell, John R. B. Perry, Olli Raitakari, Fernando Rivadeneira, Seang-Mei Saw, Sylvain Sebert, John A. Shepherd, Marie Standl, Thorkild I. A. Sørensen, Nicholas J. Timpson, Maties Torrent, Gonneke Willemsen, Elina Hypponen, Chris Power, The Early Growth Genetics Consortium, Mark I. McCarthy, Rachel M. Freathy, Elisabeth Widén, Hakon Hakonarson, Inga Prokopenko, Benjamin F. Voight, Babette S. Zemel, Struan F. A. Grant, Diana L. Cousminer

**Affiliations:** 1 Center for Applied Genomics, Children’s Hospital of Philadelphia, Philadelphia, PA 19104 USA; 2 Center for Spatial and Functional Genomics, Children’s Hospital of Philadelphia, Philadelphia, PA 19104 USA; 3 Department of Psychiatry, Perelman School of Medicine, University of Pennsylvania, Philadelphia, USA; 4 Department of Clinical & Experimental Medicine, University of Surrey, Guildford, UK; 5 Department of Metabolism, Digestion and Reproduction, Imperial College London, London, UK; 6 People-Centred Artifcial Intelligence Institute, University of Surrey, Guildford, UK; 7 Department of Health Research Methods, Evidence, and Impact, McMaster University, Hamilton, Canada; 8 Division of Chronic Disease Research Across the Lifecourse, Department of Population Medicine, Harvard Medical School and Harvard Pilgrim Health Care Institute, Boston, MA 02215 USA; 9 MRC Integrative Epidemiology Unit at the University of Bristol, Bristol, UK; 10 Department of Genetics, University of North Carolina, Chapel Hill, NC USA; 11 Department of Population and Public Health Sciences, University of Southern California, Los Angeles, CA 90032 USA; 12 Saw Swee Hock School of Public Health, National University of Singapore and National University Health System, Singapore, Singapore; 13 Bristol Medical School, Population Health Sciences, University of Bristol, Bristol, UK; 14 K.G. Jebsen Center for Genetic Epidemiology, Department of Public Health and Nursing, NTNU, Norwegian University of Science and Technology, Trondheim, Norway; 15 ISGlobal, Barcelona, Spain; 16 Center for Life Course Health Research, University of Oulu, Oulu, Finland; 17 Department of Biological Psychology, Vrije Universiteit Amsterdam, Amsterdam, The Netherlands; 18 Department of Statistics and Computational Research, Universitat de València, Valencia, Spain; 19 CIBER Epidemiología y Salud Pública (CIBERESP), Madrid, Spain; 20 Epidemiology and Environmental Health Joint Research Unit, FISABIO-Universitat Jaume I-Universitat de València, Valencia, Spain; 21 Phoenix Epidemiology and Clinical Research Center, NIDDK, NIH, Bethesda, USA; 22 Institute for Molecular Medicine Finland, University of Helsinki, Helsinki, Finland; 23 Department of Clinical Physiology, Finnish Cardiovascular Research Center - Tampere, Faculty of Medicine and Health Technology, Tampere University, 33014 Tampere, Finland; 24 Department of Clinical Physiology, Tampere University Hospital, 33521 Tampere, Finland; 25 Wellcome Centre for Human Genetics, University of Oxford, Oxford, OX3 7BN UK; 26 Research Centre of Applied and Preventive Cardiovascular Medicine, University of Turku, Turku, Finland; 27 Centre for Population Health Research, University of Turku and Turku University Hospital, Turku, Finland; 28 Faculty of Health and Medical Sciences, Novo Nordisk Foundation Center for Basic Metabolic Research, University of Copenhagen, Copenhagen, Denmark; 29 Population Health Research Institute, St George’s, University of London, Cranmer Terrace, London, SW17 0RE UK; 30 Institute of Epidemiology, Helmholtz Zentrum München- German Research Center for Environmental Health, Neuherberg, Germany; 31 Division of Metabolic and Nutritional Medicine, Dr. Von Hauner Children’s Hospital, University of Munich Medical Center, Munich, Germany; 32 The Generation R Study Group, Erasmus MC, University Medical Center Rotterdam, Rotterdam, the Netherlands; 33 Department of Epidemiology, Erasmus MC, University Medical Center Rotterdam, Rotterdam, the Netherlands; 34 Department of Pediatrics, Erasmus MC, University Medical Center Rotterdam, Rotterdam, the Netherlands; 35 School of Medicine and Public Health, Faculty of Medicine and Health, University of Newcastle, Callaghan, NSW 2308 Australia; 36 Hunter Medical Research Institute, Newcastle, NSW 2305 Australia; 37 MRC Unit for Lifelong Health and Ageing at UCL, London, UK; 38 Department of Pediatrics, The Children’s Obesity Clinic, Copenhagen University Hospital Holbæk, Holbæk, Denmark; 39 Department of Pathology and Laboratory Medicine, Children’s Hospital of Philadelphia, Philadelphia, PA USA; 40 Faculty of Health and Medical Sciences, University of Western Australia, Perth, WA Australia; 41 Unidad de Investigación Médica en Bioquímica, Hospital de Especialidades, Centro Médico Nacional Siglo XXI, Instituto Mexicano del Seguro Social, Mexico City, Mexico; 42 MRC Centre for Environment and Health, School of Public Health, Faculty of Medicine, Imperial College London, St Mary’s Campus, Norfolk Place, London, W2 1PG UK; 43 Institute of Reproductive & Developmental Biology, Imperial College London, London, UK; 44 Center for Endocrinology, Diabetes & Metabolism, Children’s Hospital Los Angeles, Los Angeles, CA USA; 45 Haverford College, Haverford, PA USA; 46 Department of Pediatrics, Cincinnati Children’s Hospital, University of Cincinnati, Cincinnati, OH USA; 47 Department of Pediatrics, The University of Pennsylvania Perelman School of Medicine, Philadelphia, PA 19104 USA; 48 Division of Endocrinology & Diabetes, Children’s Hospital of Philadelphia, Philadelphia, PA 19104 USA; 49 College of Family and Consumer Sciences, University of Georgia, Athens, GA USA; 50 Singapore Eye Research Institute, Singapore National Eye Centre, Singapore, Singapore; 51 Department of Medicine and College of Nursing, Creighton University School of Medicine, Omaha, NB USA; 52 USC-Ofce of Population Studies Foundation, Inc, University of San Carlos, Cebu, Philippines; 53 Division of Gastroenterology, Hepatology and Nutrition, The Children’s Hospital of Philadelphia, Philadelphia, PA 19104 USA; 54 Department of Clinical Chemistry, Faculty of Medicine and Health Technology, Finnish Cardiovascular Research Center - Tampere, Tampere University, 33014 Tampere, Finland; 55 Department of Clinical Chemistry, Fimlab Laboratories, 33520 Tampere, Finland; 56 Department of Pediatrics and Adolescent Medicine, Turku University Hospital and University of Turku, Turku, Finland; 57 Department of Physiology, University of Turku, Turku, Finland; 58 Department of Nutrition, Harvard T.H Chan School of Public Health, Boston, MA 02115 USA; 59 Paavo Nurmi Centre, Unit for Health and Physical Activity, University of Turku, Turku, Finland; 60 Department of Nutrition, Gillings School of Global Public Health, University of North Carolina, Chapel Hill, NC USA; 61 Amsterdam Reproduction & Development (AR&D) Research Institute, Amsterdam, the Netherlands; 62 Universitat Pompeu Fabra (UPF), Barcelona, Spain; 63 Institute of Clinical Medicine Department of General Practice and Primary Health Care, University of Helsinki, Helsinki, Finland; 64 Folkhälsan Research Center, Helsinki, Finland; 65 Department of Obstetrics & Gynecology, Yong Loo Lin School of Medicine, National University of Singapore, Singapore, Singapore; 66 Cohort and Longitudinal Studies Enhancement Resources (CLOSER), UCL Institute of Education, London, UK; 67 The Faculty of Health and Medical Sciences, University of Copenhagen, Copenhagen, Denmark; 68 Department of Epidemiology and Biostatistics, School of Public Health, MRC-PHE Centre for Environment and Health, Imperial College London, London, W2 1PG UK; 69 Unit of Primary Health Care, Oulu University Hospital, OYS, Kajaanintie 50, 90220 Oulu, Finland; 70 Lions Eye Institute, Centre for Ophthalmology and Visual Science, Centre for Eye Research Australia, University of Western Australia, Perth, WA Australia; 71 Department of Pathology and Molecular Medicine, McMaster University, Hamilton, Canada; 72 Inserm UMR_S1256 Nutrition-Genetics-Environmental Risk Exposure, University of Lorraine, Nancy, France; 73 Department of Biochemistry-Molecular Biology-Nutrition, University Hospital Centre of Nancy, Nancy, France; 74 Division of Pediatric Endocrinology, Columbia University Medical Center, New York, NY USA; 75 Department of Maternity and Gynaecology, John Hunter Hospital, Newcastle, NSW 2305 Australia; 76 Metabolic Research Laboratory, School of Clinical Medicine, Wellcome-MRC Institute of Metabolic Science, University of Cambridge, Cambridge, CB2 0QQ UK; 77 MRC Epidemiology Unit, School of Clinical Medicine, Wellcome-MRC Institute of Metabolic Science, University of Cambridge, Cambridge, CB2 0QQ UK; 78 Department of Clinical Physiology and Nuclear Medicine, Turku University Hospital, Turku, Finland; 79 Department of Internal Medicine, Erasmus MC, University Medical Center Rotterdam, Rotterdam, the Netherlands; 80 Department of Epidemiology and Population Science, University of Hawaii Cancer Center, Honolulu, HI USA; 81 Department of Public Health, Faculty of Health and Medical Sciences, University of Copenhagen, Copenhagen, Denmark; 82 Fundació Institut d’Investigació Sanitària Illes Balears – IdISBa, Palma, Spain; 83 UCL Great Ormond Street Institute of Child Health, London, UK; 84 Australian Centre for Precision Health, Unit of Clinical and Health Sciences, University of South Australia, Adelaide, Australia; 85 South Australian Health and Medical Research Institute, Adelaide, Australia; 86 Department of Clinical and Biomedical Sciences, Faculty of Health and Life Sciences, University of Exeter, Exeter, EX2 5DW UK; 87 UMR 8199 - EGID, Institut Pasteur de Lille, CNRS, University of Lille, 59000 Lille, France; 88 Department of Genetics, University of Pennsylvania, Philadelphia, PA 19104 USA; 89 Department of Systems Pharmacology and Translational Therapeutics, Perelman School of Medicine, University of Pennsylvania, Philadelphia, PA 19104 USA; 90 Institute of Translational Medicine and Therapeutics, University of Pennsylvania, Philadelphia, PA 19104 USA; 91 Division of Human Genetics, Children’s Hospital of Philadelphia, Philadelphia, PA 19104 USA; 92 Currently Employed By GlaxoSmithKline, 1250 S Collegeville Rd, Collegeville, PA 19426 USA; 93 Current Address: Genentech, 1 DNA Way, San Francisco, CA 94080 USA

**Correction** **:** **Genome Biol 25, 22 (2024)**

**https://doi.org/10.1186/s13059-023-03136-z**

Following publication of the original article [1], the authors identified an error in the author name of Zhanna Balkhiyarova.

The incorrect author name is: Zhanna Balkiyarova

The correct author name is: Zhanna Balkhiyarova

The author group has been updated above and the original article [1] has been corrected.
