# Supplementary material for: Trans-ancestral genome-wide association study of longitudinal pubertal height growth and shared heritability with adult health outcomes
Source: Genome Biol. 2024 Jan 16;25:22. doi: 10.1186/s13059-023-03136-z (PMC10790528; doi:10.1186/s13059-023-03136-z)
Supplement: Supplementary file 2 — Additional file 2. Figures S1-S5, Cohort funding and acknowledgments. [file 13059_2023_3136_MOESM2_ESM.docx]

**Supplementary Figures**

**Fig. S1. Three example regional association plots of genome-wide**

**significant loci.**

**
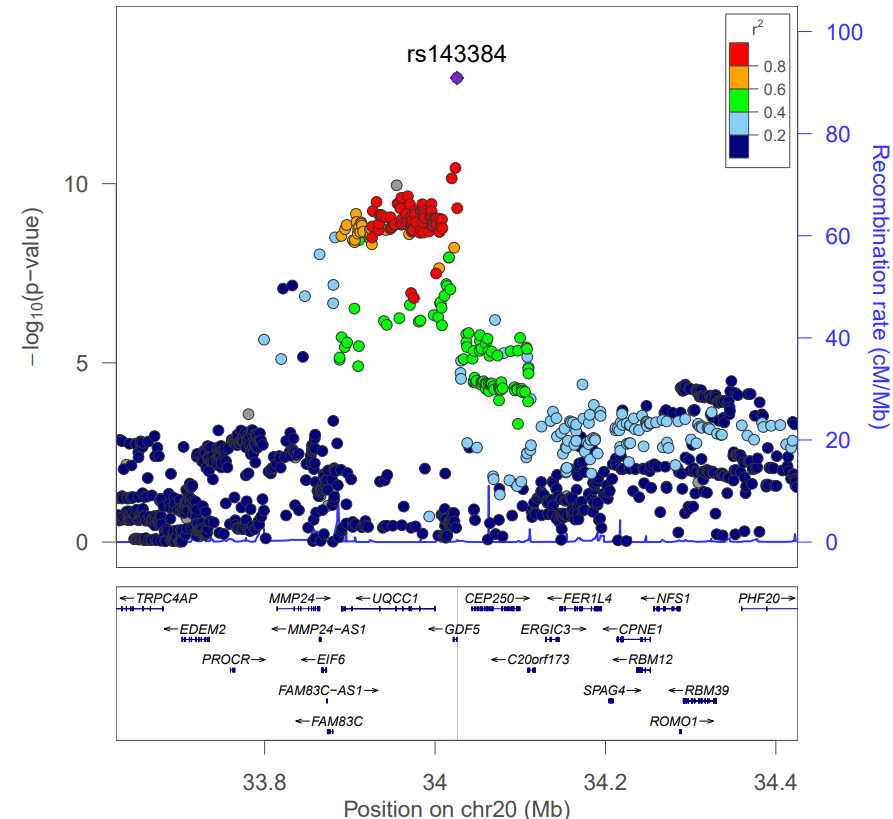
**


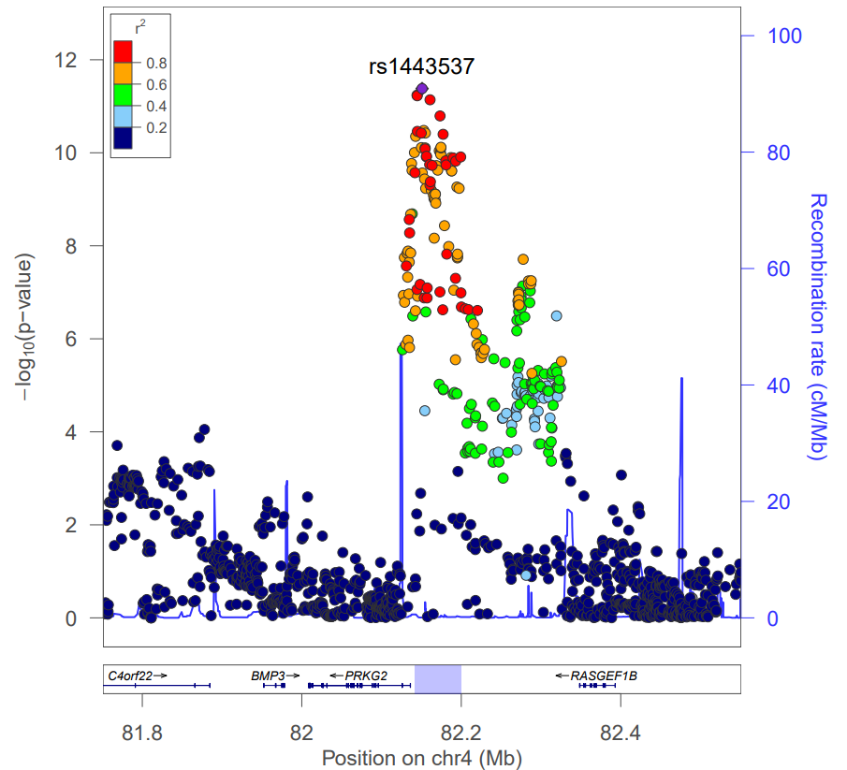


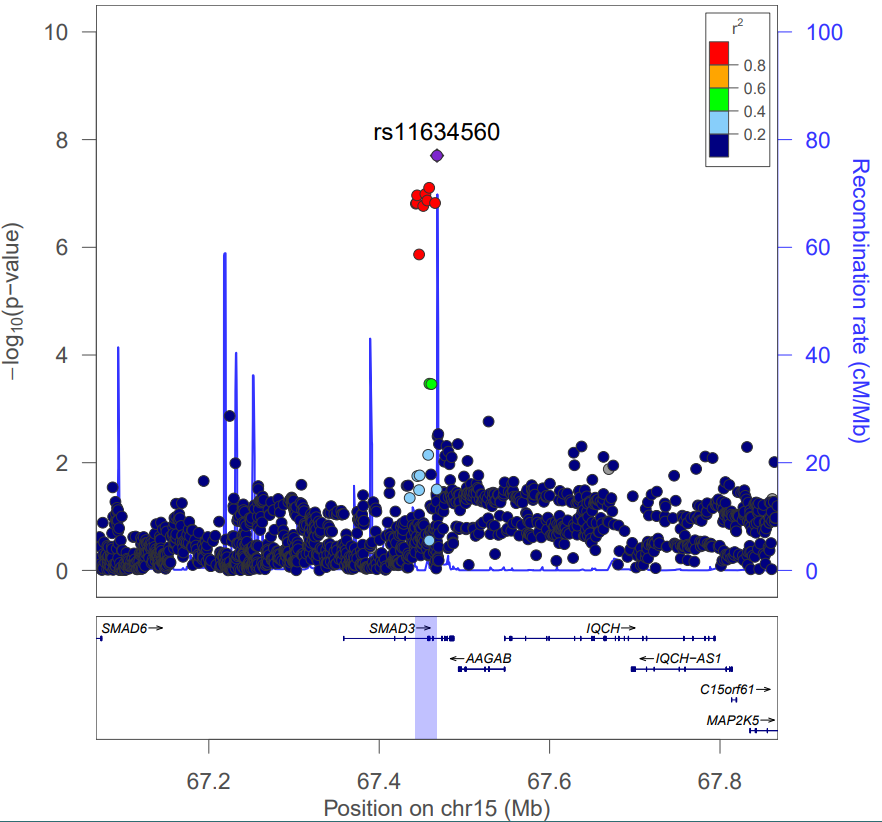


**Fig. S2. Specific loci did not cluster with the other associations.**  A) Clustering of the loci based on trans-ancestry associations with *a-size*, *b-timing*, and *c-intensity* revealed two loci with distinct association patterns, at the *LIN28B* and *HBB* loci. Points are plotted based on their association values with the three SITAR phenotypes and are color-coded by their genome-wide significant phenotype as shown in the legend. For instance, the *HBB* locus was significant for phenotype V, while the *LIN28B* phenotype showed significant associations with phenotypes III, V, and VI. B) Association of the *HBB* locus in the Children’s Hospital of Philadelphia Center for Applied Genomics (CAG) cohort. C) Association in the CAG after conditioning on sickle cell anemia status.

**A.**


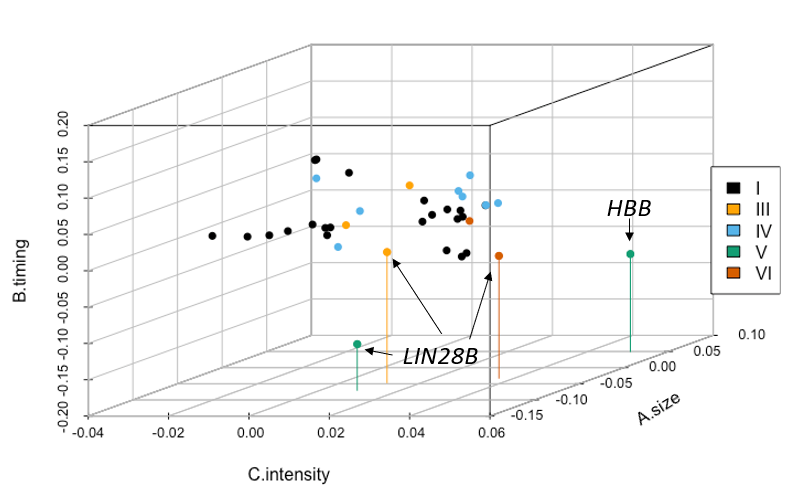


**B.**

**
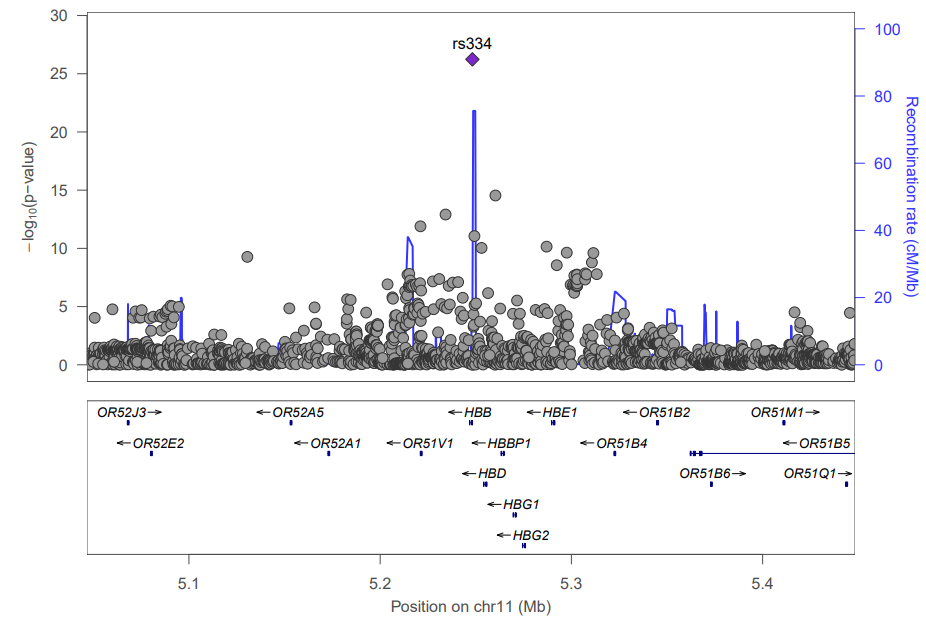
**

**C.**


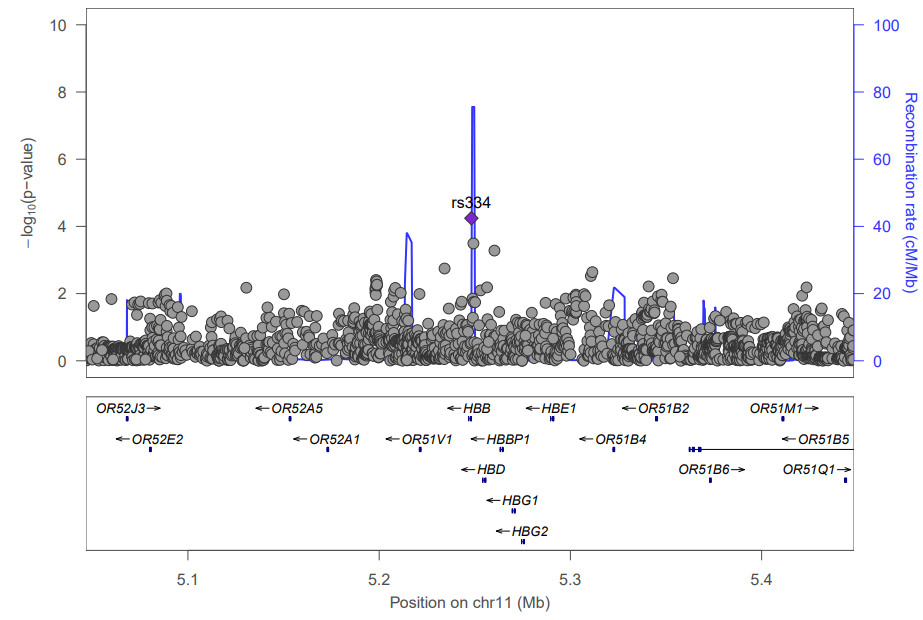


**Fig. S3. Genetic correlations between pubertal growth phenotypes using LD Score Regression analysis.** Full data shown in Supplementary Table 7. 10F/12M, height at age 10 years in girls and age 12 years in boys; 14-adult, height difference between age 14 years and adult; 8-adult, height difference between age 8 years and adult; a-size, SITAR-derived height across the growth trajectory; c-intensity, SITAR-derived tempo of the pubertal growth spurt.


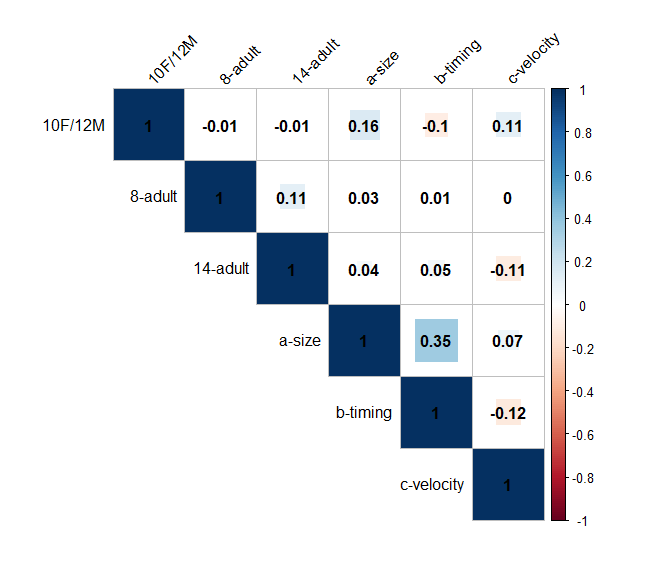


**Fig. S4. Manhattan plots.** Credible set SNPs are highlighted in yellow.

**
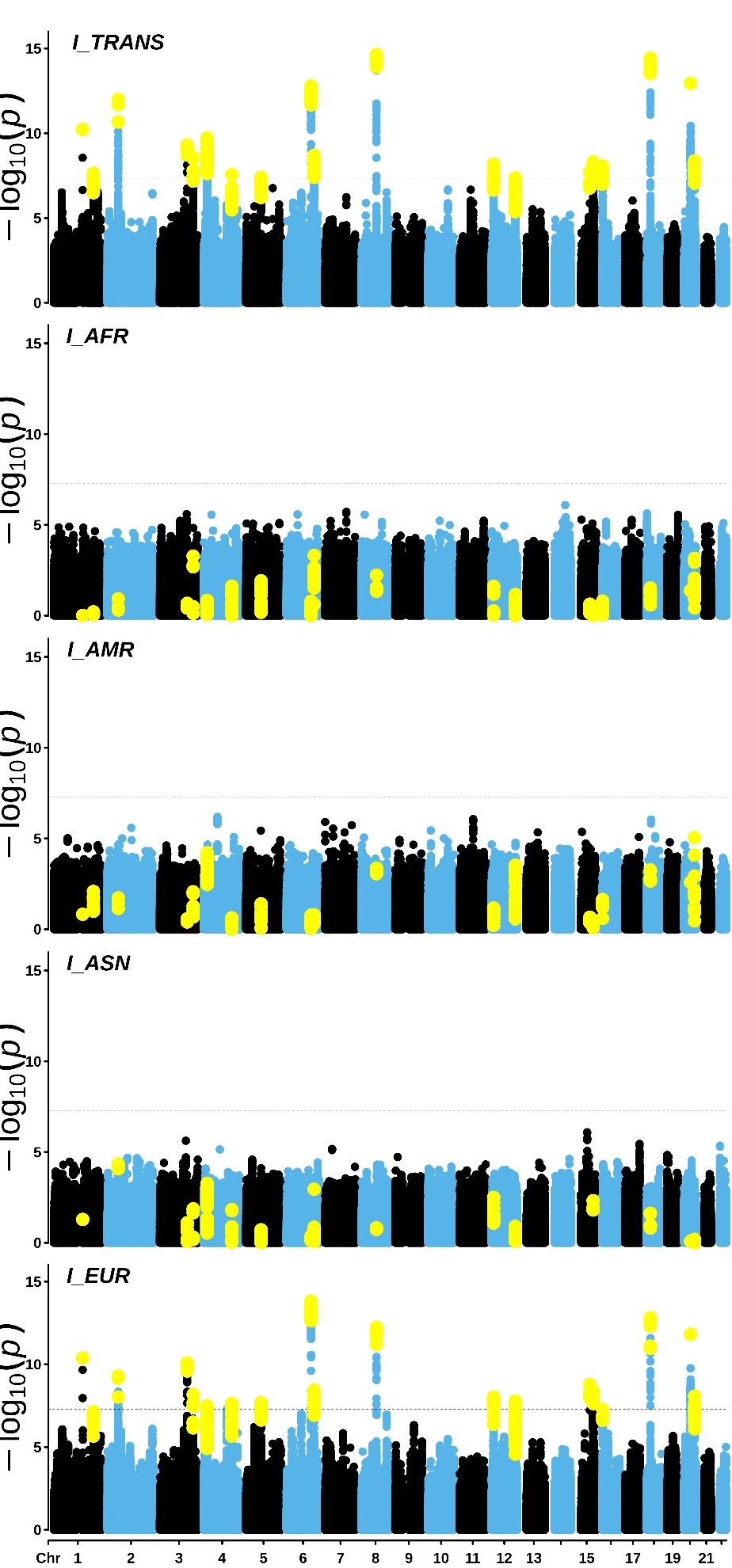

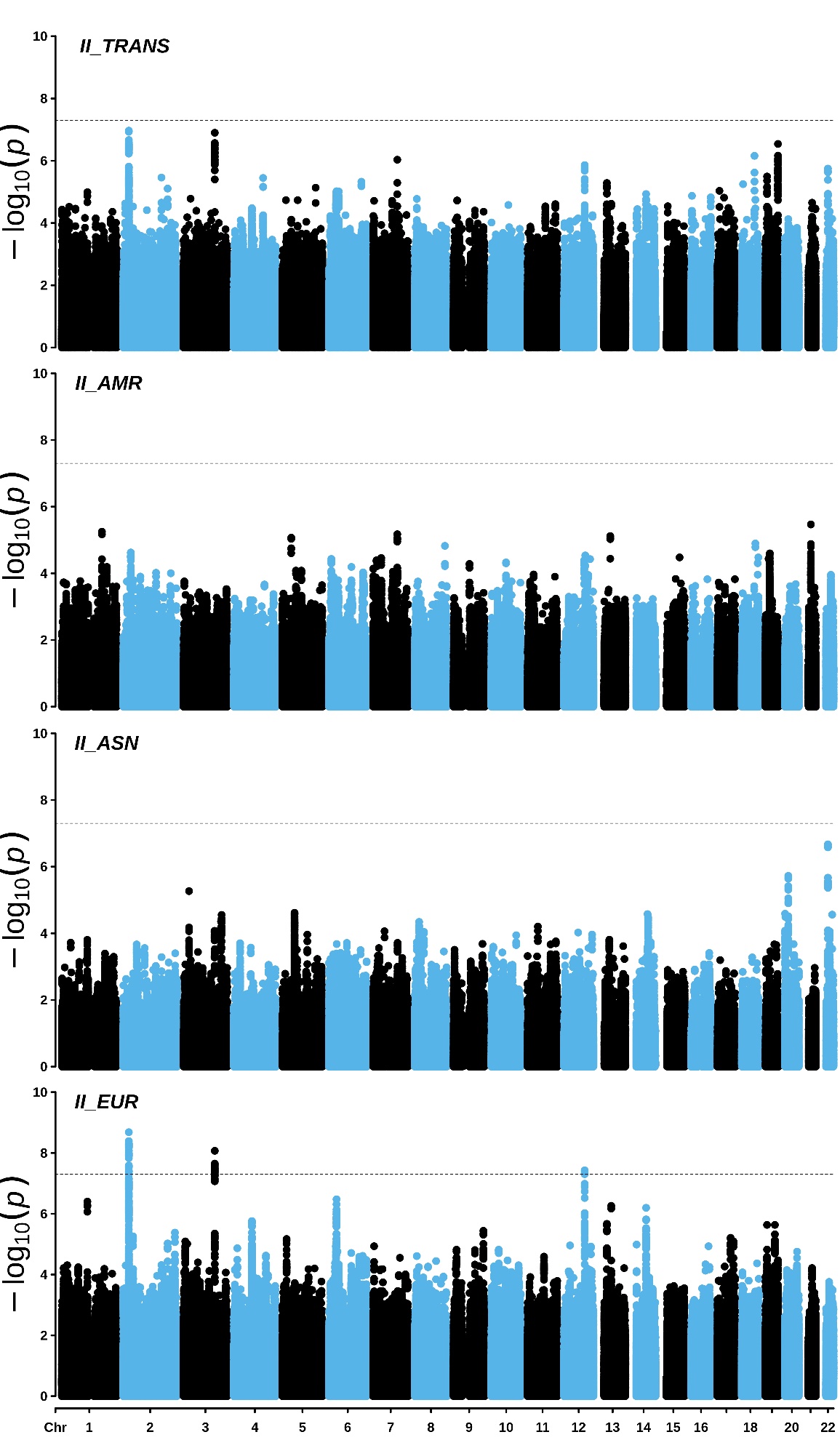

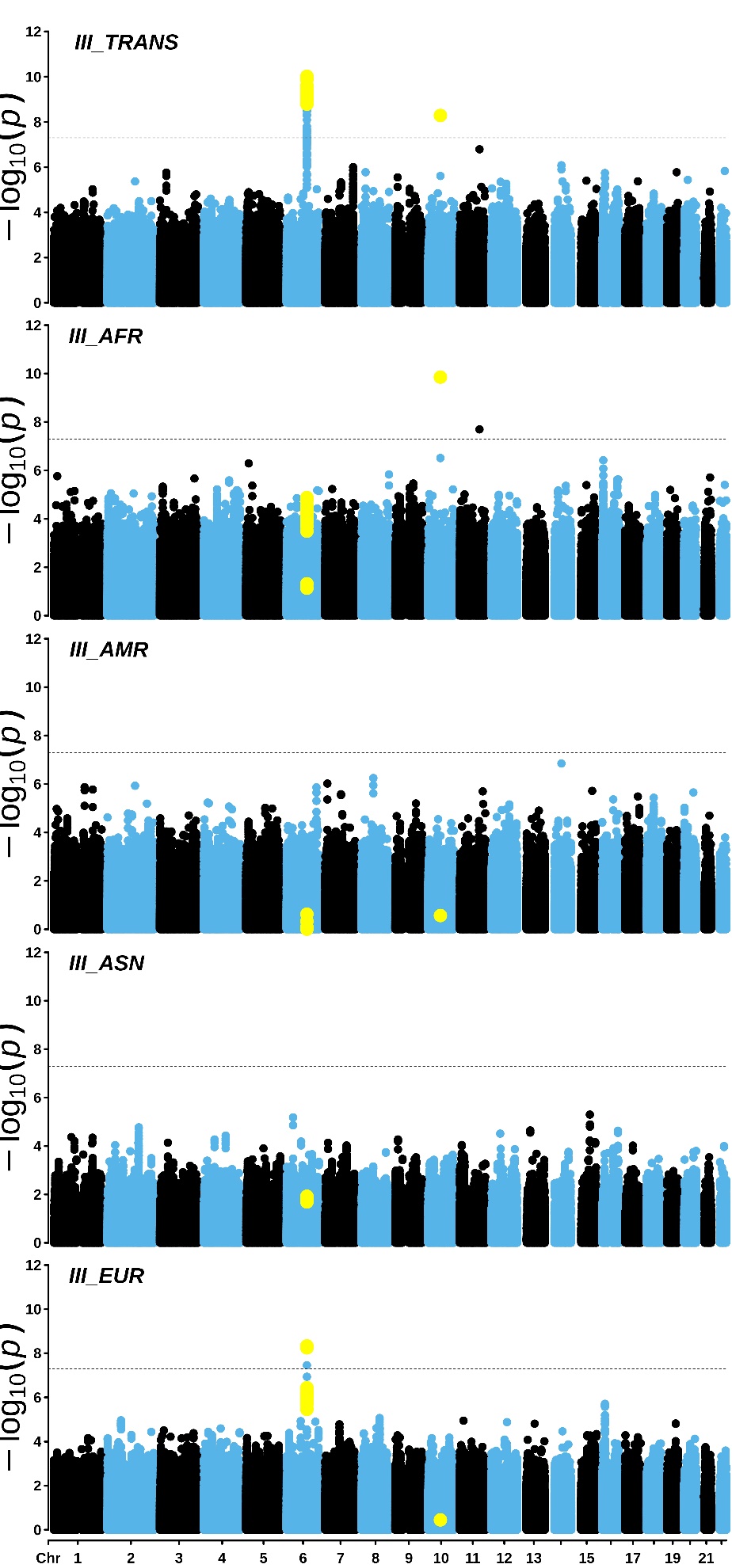

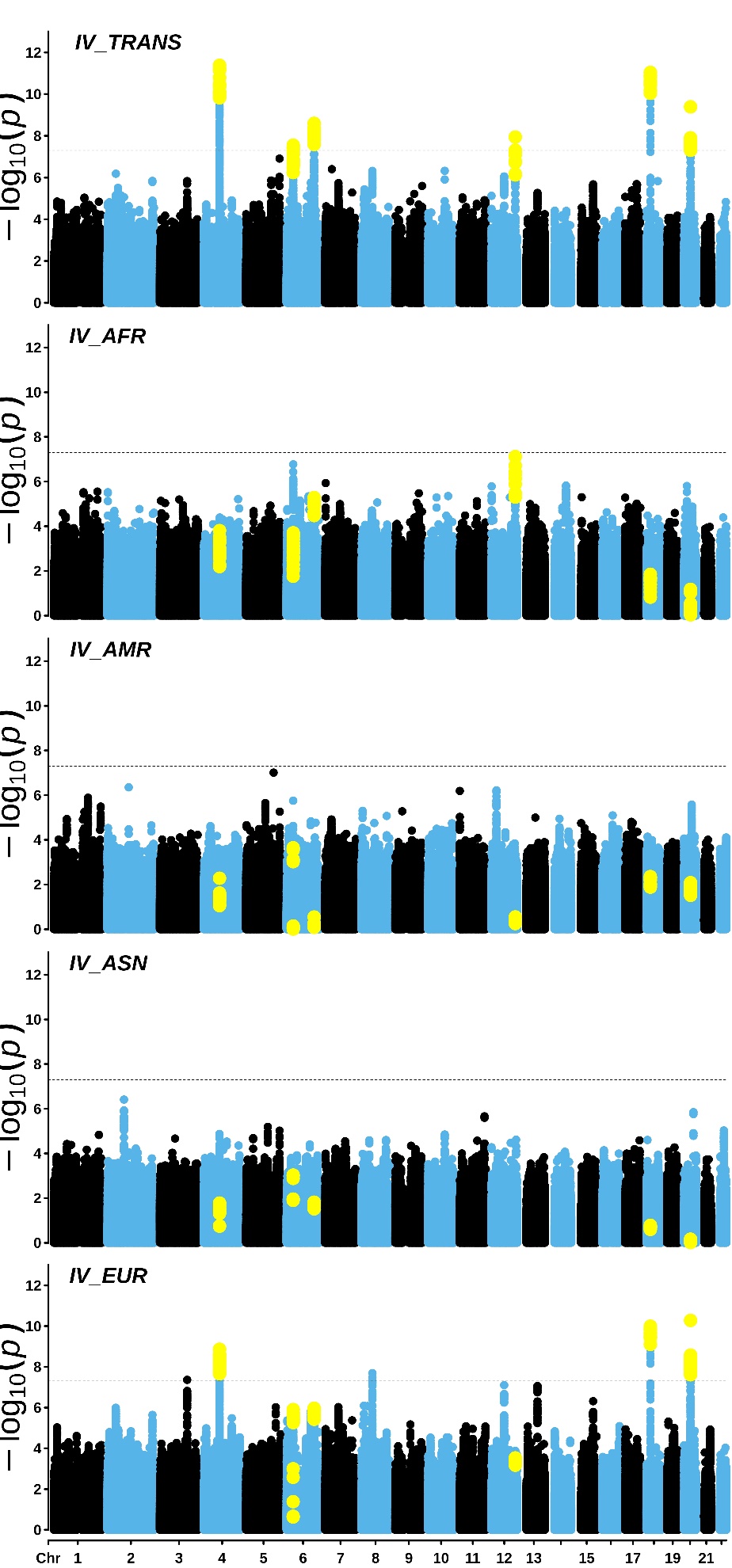

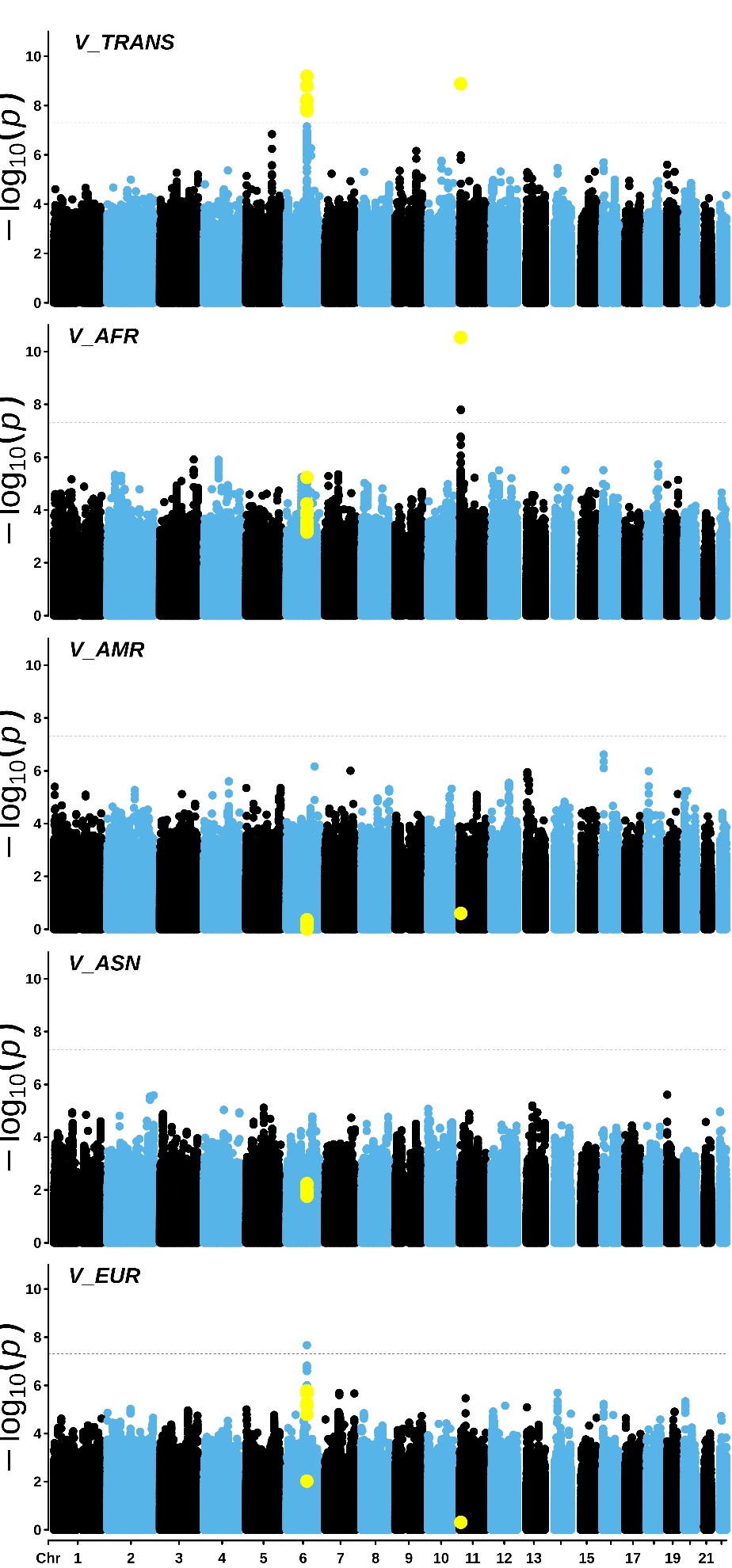

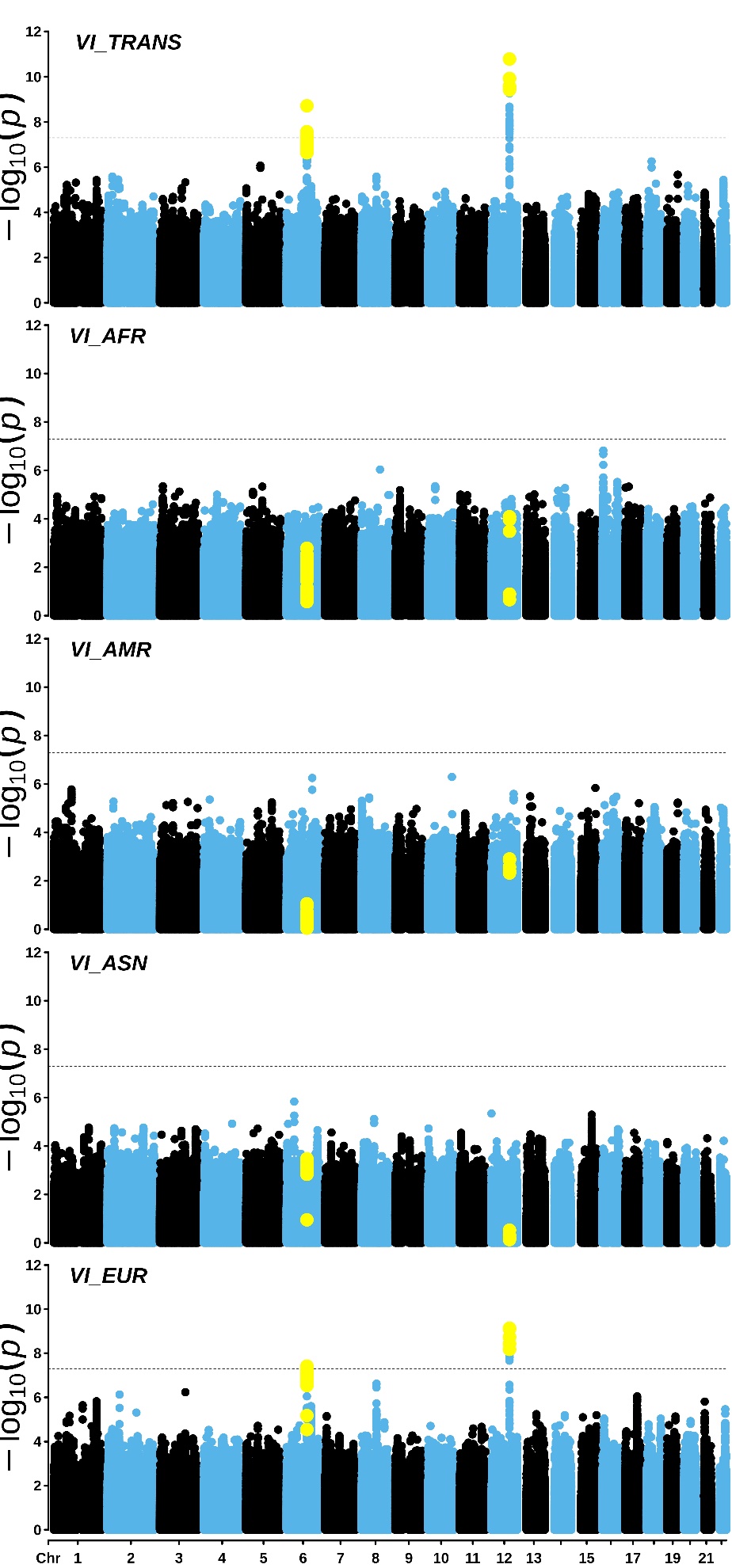
**

**Fig. S5. QQ plots.**

**
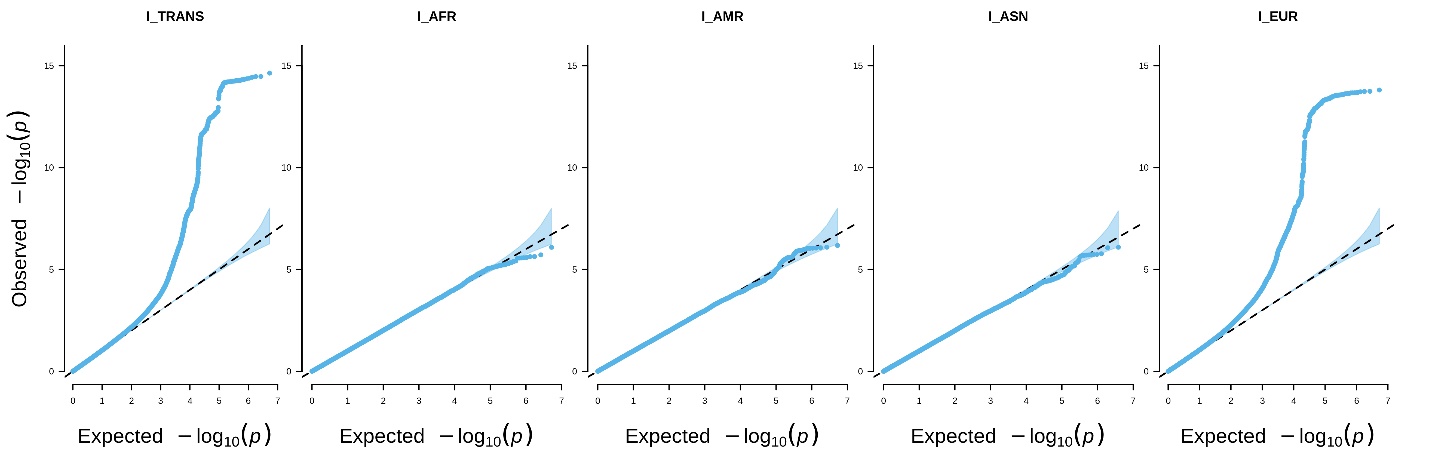

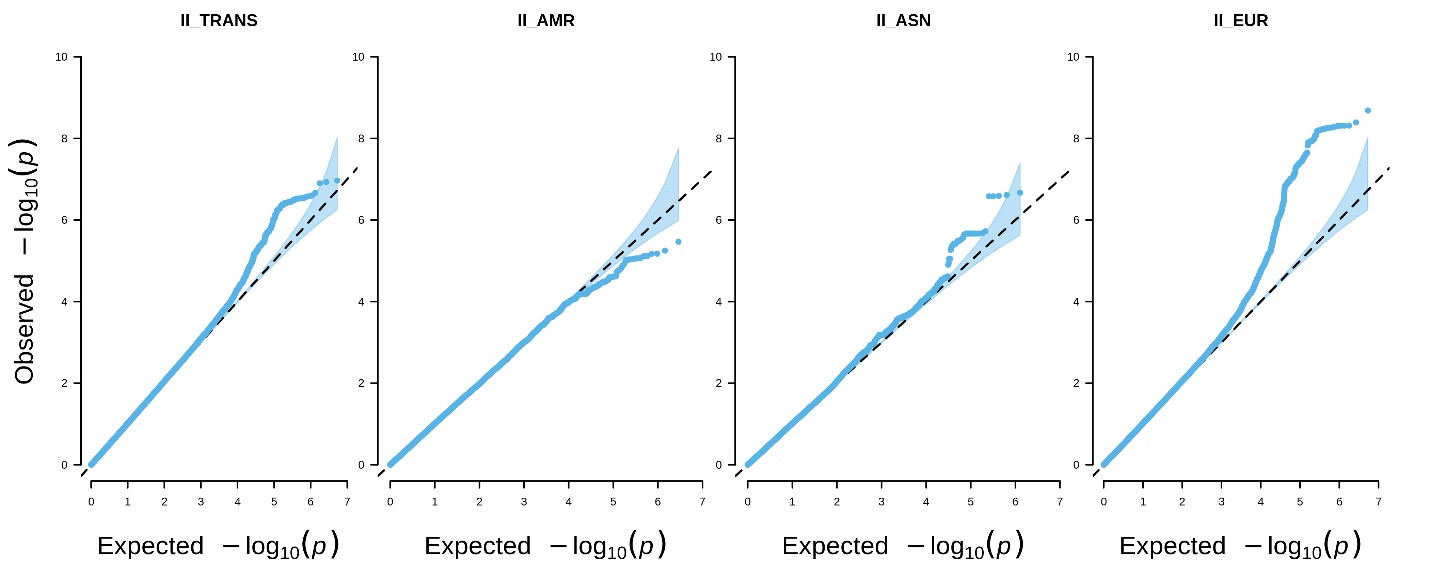

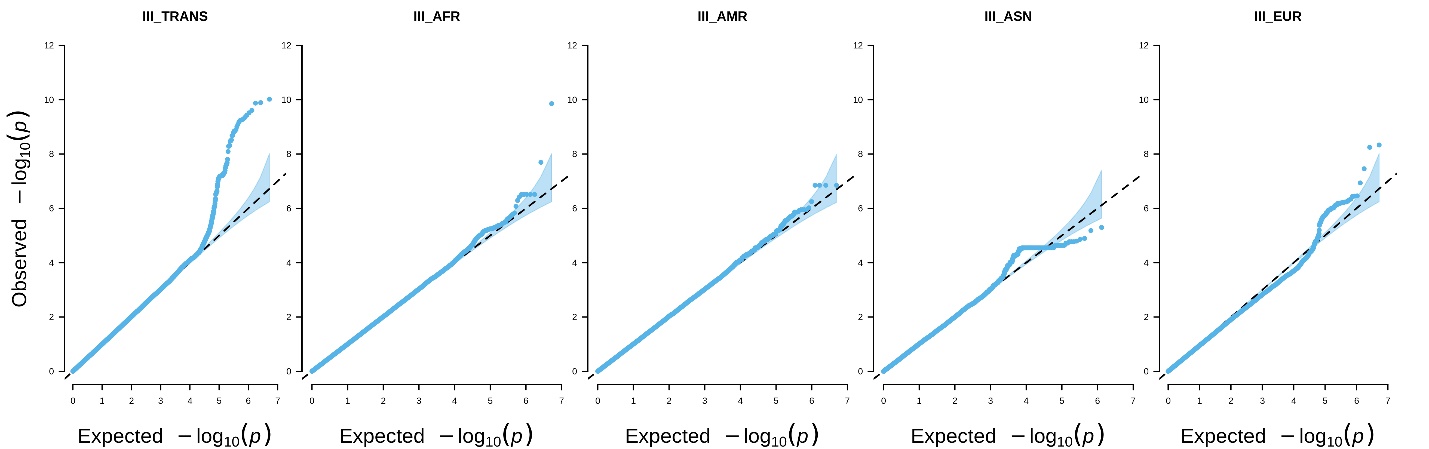

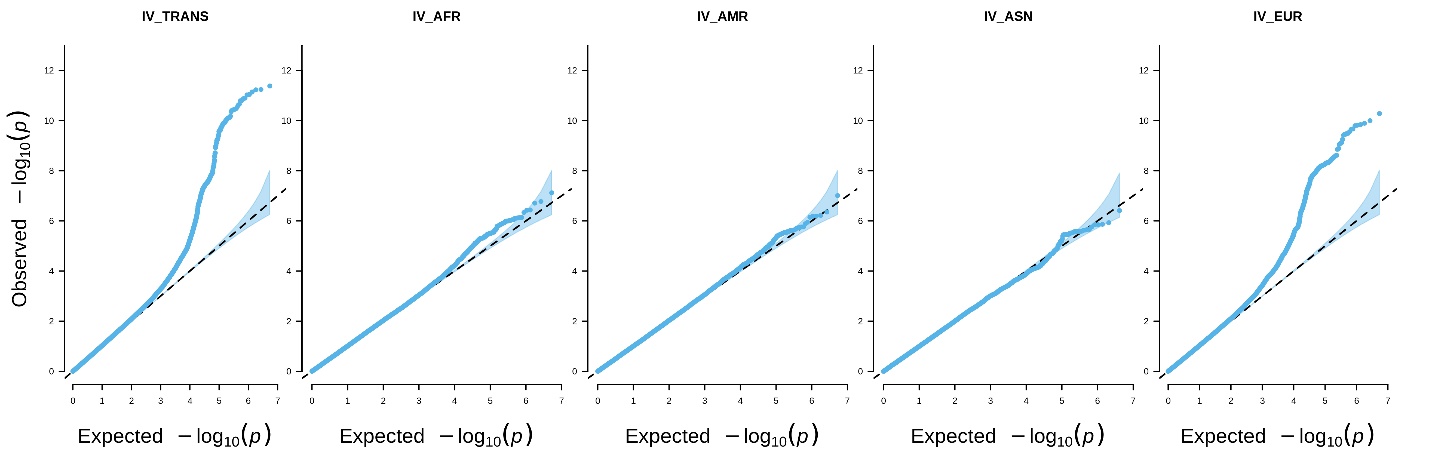

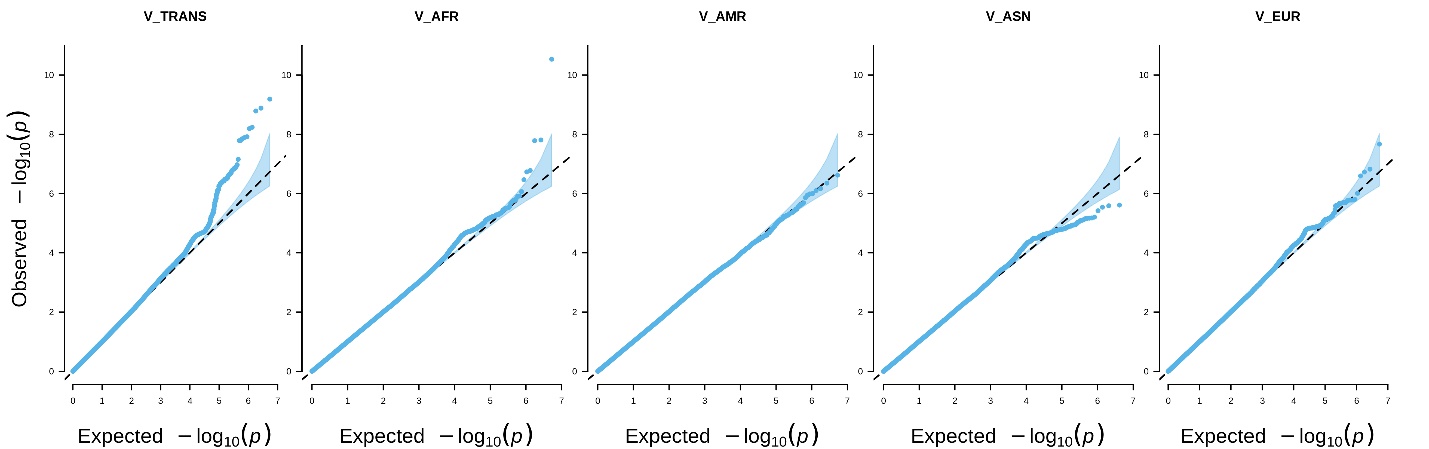

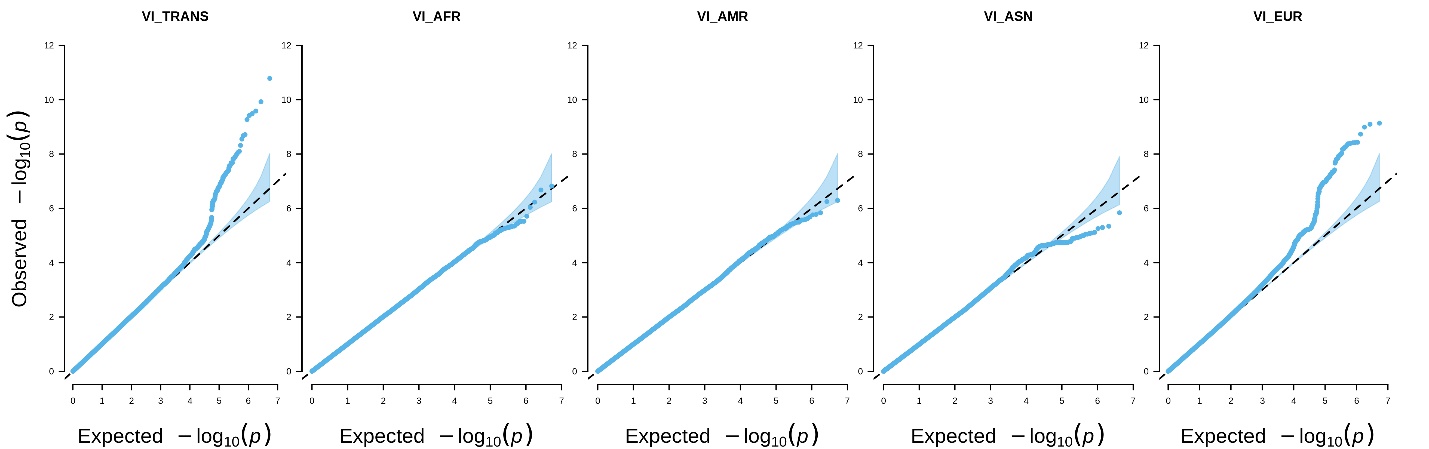
**

**Cohort funding and acknowledgments**

The MRC National Survey of Health and Development is funded by the Medical Research Council (MC_UU_10019/1).

This research has been conducted using the UK Biobank Resource under Application Number 37685.

The authors thank all the NSHD participants for both their contributions and for their commitments to medical research.

The Cebu Longitudinal Health and Nutrition Survey (CLHNS) was supported by National Institutes of Health grants DK078150, TW005596, HL085144 and TW008288 and pilot funds from RR020649, ES010126, and DK056350. We thank the Office of Population Studies Foundation research and data collection teams and the study participants who generously provided their time for this study.

The Raine Study was supported by the National Health and Medical Research Council of Australia [grant numbers 572613, 403981 and 1059711] and the Canadian Institutes of Health Research [grant number MOP-82893].

The authors are grateful to the Raine Study participants and their families, and to the Raine Study research staff for cohort coordination and data collection. The authors gratefully acknowledge the NH&MRC for their long term funding to the study over the last 30 years and also the following institutes for providing funding for Core Management of the Raine Study: The University of Western Australia (UWA) , Curtin University, the Raine Medical Research Foundation, the UWA Faculty of Medicine, Dentistry and Health Sciences, the Telethon Kids Institute, the Women's and Infant's Research Foundation (King Edward Memorial Hospital), Murdoch University, The University of Notre Dame (Australia), and Edith Cowan University. The authors gratefully acknowledge the assistance of the Western Australian DNA Bank (National Health and Medical Research Council of Australia National Enabling Facility). We would also like to acknowledge the Raine Study participants for their ongoing participation in the study, and the Raine Study Team for study co-ordination and data collection. This work was supported by resources provided by the Pawsey Supercomputing Centre with funding from the Australian Government and Government of Western Australia.

Special Turku Coronary Risk Factor Intervention Project (STRIP) has been financially supported by Academy of Finland (grants 206374, 251360 and 276861); Juho Vainio Foundation; Finnish Cardiac Research Foundation; Finnish Cultural Foundation; Finnish Ministry of Education and Culture; Sigrid Juselius Foundation; Yrjö Jahnsson Foundation; C.G. Sundell Foundation; Special Governmental Grants for Health Sciences Research, Turku University Hospital; Foundation for Pediatric Research; and Turku University Foundation.

We acknowledge the Penn Medicine BioBank (PMBB) for providing data and thank the patient-participants of Penn Medicine who consented to participate in this research program. We would also like to thank the Penn Medicine BioBank team and Regeneron Genetics Center for providing genetic variant data for analysis.  The PMBB is approved under IRB protocol# 813913 and supported by Perelman School of Medicine at University of Pennsylvania, a gift from the Smilow family, and the National Center for Advancing Translational Sciences of the National Institutes of Health under CTSA award number UL1TR001878.

We thank the participants of the Mexican children study for their contribution. This work was supported by the Consejo Nacional de Ciencia y Tecnología (CONACYT-México) with the grant SALUD-2013-C01-201471 (FONSECSSA/IMSS/ISSSTE). Computations were performed on the GPC supercomputer at the SciNet HPC Consortium, Canada. SciNet is funded by: the Canada Foundation for Innovation under the auspices of Compute Canada; the Government of Ontario; Ontario Research Fund-Research Excellence; and the University of Toronto.

We are extremely grateful to all the families who took part in this study, the midwives for their help in recruiting them, and the whole ALSPAC team, which includes interviewers, computer and laboratory technicians, clerical workers, research scientists, volunteers, managers, receptionists and nurses. The UK Medical Research Council and Wellcome (Grant ref: 217065/Z/19/Z) and the University of Bristol provide core support for ALSPAC. This publication is the work of the authors and DLC and SFAG will serve as guarantors for the contents of this paper. GWAS data was generated by Sample Logistics and Genotyping Facilities at Wellcome Sanger Institute and LabCorp (Laboratory Corporation of America) using support from 23andMe.

Young Finns Study (YFS) thanks the teams that collected data at all measurement time points; the persons who participated as both children and adults in these longitudinal studies; and biostatisticians Irina Lisinen, Johanna Ikonen, Noora Kartiosuo, Ville Aalto, and Jarno Kankaanranta for data management and statistical advice. The Young Finns Study has been financially supported by the Academy of Finland: grants 322098, 286284, 134309 (Eye), 126925, 121584, 124282, 255381, 256474, 283115, 319060, 320297, 314389, 338395, 330809, 104821, 129378 (Salve), 117797 (Gendi), and 141071 (Skidi); the Social Insurance Institution of Finland; Competitive State Research Financing of the Expert Responsibility area of Kuopio, Tampere and Turku University Hospitals (grant X51001); Juho Vainio Foundation; Paavo Nurmi Foundation; Finnish Foundation for Cardiovascular Research; Finnish Cultural Foundation; The Sigrid Juselius Foundation; Tampere Tuberculosis Foundation; Emil Aaltonen Foundation; Yrjö Jahnsson Foundation; Signe and Ane Gyllenberg Foundation; Diabetes Research Foundation of Finnish Diabetes Association; EU Horizon 2020 (grant 755320 for TAXINOMISIS and grant 848146 for To Aition); European Research Council (grant 742927 for MULTIEPIGEN project); Tampere University Hospital Supporting Foundation, Finnish Society of Clinical Chemistry and the Cancer Foundation Finland.

The Generation R Study is conducted by Erasmus MC in close collaboration with the School of Law and Faculty of Social Sciences of the Erasmus University Rotterdam, the Municipal Health Service Rotterdam area, Rotterdam, the Rotterdam Homecare Foundation, Rotterdam and the Stichting Trombosedienst & Artsenlaboratorium Rijnmond (STAR-MDC), Rotterdam. We gratefully acknowledge the contribution of children and parents, general practitioners, hospitals, midwives and pharmacies in Rotterdam. The study protocol was approved by the Medical Ethical Committee of Erasmus MC, Rotterdam. Written informed consent was obtained from all participants. The generation and management of GWAS genotype data for the Generation R Study were done at the Genetic Laboratory of the Department of Internal Medicine, Erasmus MC, The Netherlands. We would like to thank Karol Estrada, Dr. Tobias A. Knoch, Anis Abuseiris, Luc V. de Zeeuw, and Rob de Graaf, for their help in creating GRIMP, BigGRID, MediGRID, and Services@MediGRID/D-Grid, (funded by the German Bundesministerium fuer Forschung und Technology; grants 01 AK 803 A-H, 01 IG 07015 G) for access to their grid computing resources. We thank Mila Jhamai, Manoushka Ganesh, Pascal Arp, Marijn Verkerk, Lizbeth Herrera and Marjolein Peters for their help in creating, managing and QC of the GWAS database. Also, we thank Karol Estrada for their support in creation and analysis of imputed data.

The general design of Generation R Study is made possible by financial support from Erasmus MC, Rotterdam, Erasmus University Rotterdam, the Netherlands Organization for Health Research and Development (ZonMw), the Netherlands Organisation for Scientific Research (NWO), the Ministry of Health, Welfare and Sport and the Ministry of Youth and Families. This project has received funding from the European Union’s Horizon 2020 research and innovation programme (733206, LifeCycle; 874739, LongITools; 874583, ATHLETE; 824989, EUCAN-Connect).

This research was funded in part by the Wellcome Trust [WT220390]. For the purpose of open access, the authors have applied a CC BY public copyright licence to any Author Accepted Manuscript version arising from this submission.
