# Supplementary material for: Trans-ancestral genome-wide association study of longitudinal pubertal height growth and shared heritability with adult health outcomes
Source: Genome Biol. 2024 Jan 16;25:22. doi: 10.1186/s13059-023-03136-z (PMC10790528; doi:10.1186/s13059-023-03136-z)
Supplement: Supplementary file 3 — Additional file 3. Peer review history [file 13059_2023_3136_MOESM3_ESM.docx]

**Review history**

**First round of review**

**Reviewer 1**

Comments on "Trans-ancestral genome-wide association study of longitudinal pubertal height growth and shared heritability with adult health outcomes"
The paper describes an impressive meta-analysis of longitudinal height data, analysed using the SITAR growth curve model (full disclosure - I invented it). This summarises individual growth patterns in terms of three random effects, which the authors then subjected to genetic analysis to identify genome-wide loci relating to them, and to explore the impact of these variants on later health. My comments relate largely to the SITAR analysis.
1. The three SITAR random effects, as first described in Cole et al (2010), were called size, tempo and velocity, where size is a measure of adult height, tempo indicates the age at peak height velocity (APHV), and velocity is a multiplier shrinking or stretching the age scale that corresponds to peak height velocity (PHV). The three random effects are centred so they measure how individuals differ from the mean.
The random effects each have a direct biological meaning, so it's important that their names clearly describe what that meaning is. It has turned out that 'tempo' and 'velocity' are intrinsically ambiguous - I used tempo to mean the timing of peak velocity, but others, including these authors, have used it to mean the rate of passage through puberty. See for example lines 357-8: "The relationship between pubertal timing (e.g. its onset) and tempo (e.g. the speed of progression through puberty) remains controversial." I used velocity to measure the speed of progression, but that too has not been ideal. For these reasons I stopped using tempo and velocity some years ago, and instead recommend the names timing and intensity.
The authors have shifted half-way, from tempo/velocity to timing/velocity (see line 300), but they also use tempo as a synonym for velocity, which to my mind is the worst of all worlds. The purpose of this long preamble is to urge the authors to use the terms timing and intensity, and not to use tempo at all (though they could explain why they are avoiding it).
2. I shall follow my own advice and avoid tempo from now on. The genetic results for intensity are interesting and novel, but it is really surprising that there are no equivalent results for timing, as discussed at the foot of page 11. The substantial analysis fails to find a genetic component for heritability in the timing of puberty. This is a major weakness of the analysis in that it cannot detect what is known to be a strongly heritable trait - see lines 462-3: "In our data, the b-timing parameter was not heritable, although the timing of puberty is well-established as a heritable trait."
The authors show little curiosity as to why this should be, but the most likely culprit is the quality of the data used in the SITAR analyses. It needs to be properly discussed why such a strong association should have been missed.
3. The second major point is the correlation between timing and intensity. Figure 1 shows a dramatically strong positive correlation between PHV and APHV, which on closer inspection is seen to be due to the sex difference. Supplementary Figure 1 with the same data does a much better job of showing a weaker positive association adjusted for sex, and it should replace Figure 1. However, elsewhere in the paper there is equally strong evidence for a negative correlation between timing and intensity, and this contradiction is not brought out.
Biologically one would expect the correlation to be negative. The area under the velocity curve (e.g. Figure 2) indicates the corresponding height accrued, so for individuals with the same final height, those with a later puberty will on average have grown more slowly - they have more time to grow. This generates the negative correlation, and the growth curve modelling should take this into account (see next point). The paper itself reports (lines 360-2) "a highly significant negative genetic correlation between c- velocity and age at menarche (rg = -0.52, P = 6.72x10-23), indicating that genetic determinants of later pubertal onset also favor a slower tempo of growth."
So the positive correlation in (Supplementary) Figure 1 is a puzzle, and it deserves more discussion as to what it might mean. The statement justifying it on page 11 is plain wrong: "cohorts established earlier (more historic) tended to have a later APHV and higher PHV, consistent with the secular trend toward advancing pubertal onset in the 20th century." It is true that earlier cohorts had a later APHV, but they also had a lower PHV, consistent with their being later and also shorter as adults. Perhaps sample selection is relevant, with the correlation negative within samples but positive between samples, or it might be another deficiency of the SITAR analysis, perhaps related to the noisy timing results.
4. The description of the SITAR analysis is poor, consisting of the single sentence (line 536) "Each cohort individually modeled height growth using SITAR following a standard protocol." The protocol is not even cited, nor the associated R code, and there are no summary results for the models - not even in the Supplementary Material. This lack of information is troubling, and it supports my feeling that the SITAR analyses were not as closely monitored as they should have been. There are several aspects of the fitting process that need to be specified, such as the degrees of freedom used for the cubic spline curve and whether or not age was log transformed. In addition markers of data quality such as the shape of the mean curve and the residual standard deviation need to be given. The correlation between timing and intensity is numerically smaller by definition when age is log transformed, so it really matters to know whether or not it was done.
5. Some more minor points. The Discussion suggests that (lines 468-9) "the timing of PHV relative to pubertal stage may differ between individuals." This is an odd idea, as APHV is widely recognised as linked directly to pubertal stage, occurring during stage 2 for girls and Tanner stage 3-4 for boys (see Marshall & Tanner, 1969 & 1970). For this reason the lack of pubertal staging is not an important limitation of the analysis.
6. The next sentence states that "… while annual height measurements give reasonable ability to approximate the timing of PHV, the modeled value is not directly measured, and is less accurate if fewer measurements are obtained around PHV." The authors could here cite my paper [Cole TJ. Optimal design for longitudinal studies to estimate pubertal height growth in individuals. Ann Hum Biol. 2018;45:314-20] which shows that annual height measurements estimate APHV to high accuracy, and just as well as 3-monthly measurements. The statement about fewer measurements around PHV being less accurate is uncited and probably wrong.
7. The Conclusions statement (lines 486-9) that "Being taller at early puberty associated with less growth across puberty; conversely, measures of growth from birth until adulthood were genetically correlated. Meanwhile, being shorter at early puberty correlated with a slower tempo of pubertal growth and later age at menarche." I think that "early puberty" here should be replaced by "age 10/12 for girls/boys" to make better sense.
8. Genetic correlations were done with "python scripts downloaded from GitHub" (line 599) - this would be more useful with a link.
9. The references are inconsistently formatted, with some as First Author et al. and others the complete reference.
10. Table 2. Please expand PRS in the title. Giving the numbers in the table with up to five significant digits makes no sense - please use an appropriate number of digits, no more no less. See my guidelines http://adc.bmj.com/cgi/content/full/archdischild-2014-307149. In particular do give thought to the presentation of P-values. Currently they are given in scientific format to two decimal places. But there is really no justification for giving P-values to more than one significant digit, and with GWAS the power of 10 is itself sufficient once it is smaller than say -10.
11. Figure 1. I've already said that this needs replacing with Supplementary Figure 1. As it stands it lacks units on the axes, the title phrase 'modeled by sex' is wrong, and the ggplot theme is inconsistent with the other figures.
12. Figure 2 can also be improved. It should start at age 5, reflecting the age range used in the analysis; the y-axis should include zero, and more appropriate versions of the velocity curves should be used, showing velocity plateauing after age 15. The sentence describing c-velocity (c-intensity) would be better referring to the green line being steeper or shallower, rather than more vertical/horizontal.
13. Figure 3A does not make clear at what age the adiposity phenotypes were measured.
14. In Figure 3B the orange and red points are hard to distinguish. I'm not sure that the P-values add much except complexity, as they simply reflect how far each point is from the vertical zero line. The genetic correlation of ~1 for a-size versus adult height would be worth pointing out, as it confirms that size is a direct estimate of adult height. What are the error bars (and elsewhere)?
15. In Figure 4, intelligence and years of schooling are very strongly correlated with a-size - again this would be worth discussing.
16. Supplementary Figure 2 has a coloured legend but no colour in the body of the plot. Is that right?
Tim Cole

**Reviewer 2**

Bradfield et al. performed a multi-ancestry GWAS of longitudinal pubertal height growth and examined the genetic correlation between these traits and various outcomes. This study expands on previous studies that examined only individuals with European ancestries by including multiple ancestry groups, which is an important advancement in GWAS. The manuscript appears analytically sound and the authors should be commended for synthesizing data across a large number of cohorts, however, this reviewer feels that much more details are needed as many sentences assume too much knowledge about pubertal height growth for a general genomics audience, more data presentation is necessary, and current data presentation should be improved for clarity.

First, as this is a multi-ancestry study, this reviewer considers it important the authors are themselves educated on the usage of race, ethnicity, and ancestry as population descriptors. The authors describe their analyses as "trans-ancestry" and "trans-ethnic" when reporting the same results in the manuscript without differentiating between the two, when the differences between these words are important to stakeholders. This reviewer recommends the authors follow the latest guidelines on reporting race, ancestry, and ethnicity as outlined here (see Chapter 2):
https://www.nationalacademies.org/our-work/use-of-race-ethnicity-and-ancestry-as-population-descriptors-in-genomics-research

This reviewer would like to see more analyses performed, or plots made, to address the following points:

1. Make Manhattan plots and QQ-plots for all performed discovery GWAS and multi-ancestry meta-analyses, and report the lambda inflation factors for each GWAS and meta-analysis before and after genomic control (if performed). These can be supplementary figures but are important for evaluating whether the GWAS meta-analyses were well done. In particular, this reviewer wants to know what each GWAS looked like before meta-analysis, and after. Standardize y-axes per trait so this reviewer can make informed comparisons, and add a horizontal line for your genome-wide significance threshold.

2. On lines 312, the authors introduce their fine-mapping results. In general, how well did fine-mapping perform? How many independent signals were detected for each GWAS locus? How many SNPs were in the credible sets with >10 SNPs? Were the lead SNPs by P-value also the lead SNPs by posterior inclusion probability? Mark these credible sets on the Manhattan plots requested in point 1.

3. On line 288, specifically, what does "more historic" mean in this context? From what this reviewer can tell, dates are only provided for the B58C and NFBC1966 cohorts. Could all cohorts be annotated by the date and this observation about "more historic" cohorts be more explicitly plotted in Fig. 1 or as a separate supplementary figure? This seems like an interesting finding across ancestry groups that is under-emphasized.

4. Starting on line 342, in the "Prioritized genes" section, what do these loci look like, and how well did fine-mapping perform? Can the authors generate LocusZoom plots demonstrating 2-3 loci as an additional Supplementary Figure? What were some of the genomic features of these likely candidate genes? Did they tend to be the closest gene to the top credible set SNP, or were any of the credible set SNPs mapping to their exons? If any of the described genes are not the closest gene to the credible set, are there any existing functional genomics datasets that can be used to link the credible set SNP to said gene? If there were Manhattan plots too, these locus could be annotated on it to provide more context on where these loci are.

5. More details are needed in the Methods. In many cases, the authors may have described the method in more detail in the Results section, but it is important for Methods sections to be detailed as well. Please clarify all following points and add them to the manuscript:

- Line 532, what age range constitutes "boys" and "girls"?
- Line 532, what are the "standard practices"?
- Line 536, what is the SITAR "standard protocol"?
- Line 541, what age is an "adult"?
- Line 542, what specifically was "previously described"?
- Line 551, add the HWE abbreviation after using Hardy-Weinberg equilibrium for the first time.
- Line 558, what were the "cohort-specific covariates"? Can you reference a supplementary table here?
- Line 559, what does "imputation quality" mean? What was the threshold?
- Line 561, why was GWAMA performed with "2 rounds of genomic control"? What does this mean and why was it done?
- Line 622, is "r^2=0.1" a typo for "r^2>0.1"?

6. More details are also needed in the Introduction, as this is where it feels like the authors are assuming too much knowledge for a general genomics audience. Please clarify all following points, or add more references, to the manuscript:

- Line 236, how influenced are characteristics of growth spurt by "both genetic and environmental factors"? Are there any numbers the authors can use to support this, perhaps from twin studies?
- Line 237, what does "advancing" pubertal timing mean?
- Line 238, what does "peak height growth velocity" actually measure?
- Line 241, list some examples of the "many adult health outcomes"
- Line 245, what are "bone outcomes"?
- Line 248, how heritable is "highly heritable"?
- Line 250-251, what is the "take-off phase of the growth spurt"?
- Line 253, how strong is "strongly correlated"?
- Line 254, be specific, how many signals are "about half"?
- Lines 256-257, what are the rs identifiers for the "independent signals at one genetic locus (near LIN28B)"?
- Line 260, define "associated", since this is a claim of statistical significance.
- Line 298, define "adult".
- Line 303, how much is "most" in the context of how twin studies suggest height growth is "most" heritable during adolescence?
- Lines 305-306, how does the "genetic component" found in this study compare to twin studies?
- Line 317, what is the "combined dataset" and how does it maximize discovery sample size?
- Line 322, add citations for "previously published GWAS efforts".
- Lines 338-339, this seems like an interesting finding. What does it mean that the signal was ablated? Does this confirm what was previously known in the literature? For example, the sentence at line 361-362 summarizing the findings of that paragraph nicely gives a takeaway message.
- Line 343, what percentage of GWAS signals being intergenic is "most"?
- Line 358, what about this is "controversial"?
- Lines 380-381, what does it mean that later age of menarche was genetically correlated with more growth from 8-adult and 14-adult? Was this expected, or was it suprising? Add a takeaway sentence.
- Lines 464-466, if gene by environment interactions could be important, why was it not studied here? Are there power calculations justifying why this collection of cohorts may be underpowered?

7. The remainder are requests for clarification or to make changes to the figures and tables.

All figures:
Make the colors colorblind friendly when comparing points against each other (i.e., Fig. 1, Fig. 3, Fig. 4, Fig. S2)

Figure 1:
- Line 286 refers to "each" contributing cohort, so provide the number of contributing cohorts per labelled ancestry group in the main text and in the Figure legend.
- Line 287, numbers are given and refer to Fig. 1, but there are no markers for the numbers in Fig. 1. Add some sort of indicator at 11.5 years for girls and 13.5 years for boys so this is clear in the Figure.

Figure 3:
- Panel A and B on the same plot with the same x-axis. Since these are being presented together, it would be helpful to be able to compare them on the same scale.
- What do the bars mean on the plot?
- Since the authors are plotting a lot of data, with different colors and shapes, the authors should consider switching from shapes to simply annotating significant points using standard nomenclature with asterisks (i.e., * p < 0.05, ** p < 0.001).

Figure 4:
- This figure is a bit overwhelming with the number of points combined with the different colors and shapes. Please reconsider the data presentation here. Could the authors consider plotting fewer points, and moving some to the supplement? Or perhaps panelling the figure by Model (these appear to be ggplot, try facets)?

Figure S1:
- What do the shaded bars represent?

Figure S2:
- Please add the panel labels.
- There are no recombination rate peaks or colors based on pairwise LD with the lead SNP. Since discovery GWAS was performed in this study, the authors should have this info. Either add the info or remove the legends.
- In the title, "the usual pattern of associations" is strangely vague. Can you be more specific?

Table 1:
- Add whether "position" is for hg19 or hg38.

All tables and supplementary tables:
- Add legends for abbreviations.

**Authors’ response to reviewers**

abc

**Second round of review**

**Reviewer 1**

The authors have responded very positively to` my comments and suggestions. There remain a few minor points.

1. Introduction. "PHV [is] the amount of height gained per year during the most rapid phase of height growth during puberty." Not so - peak velocity in SITAR is the instantaneous peak velocity, which is greater than the peak annual velocity.
2. "… the take-off phase of the growth spurt, the age at which growth velocity increases." Strictly speaking it's the age in mid-childhood when growth velocity starts to rise after falling since infancy.
3. Figure 1. The sex-specific regression lines differ in slope, which says to me that the underlying model is PHV ~ APHV * sex and not APHV ~ PHV + sex as stated. The legend does not include the regression lines. The title is the wrong way round and omits 'mean' - better would be "Mean peak height velocity versus mean age at peak height velocity …". Or better still, omit the title from the plot.
4. Figure 2 shows mean height velocity by age and sex, but the first three phenotypes relate to height or height difference, which would make more sense with plots of height by age and sex.
5. Line 311. Perhaps state explicitly that b-timing was the only one of the six phenotypes not to have a significant genetic component.
6. Line 342. "The tempo of pubertal growth … taller height". I suggest omitting the phrase "the tempo of" since the comment relates to size as well as timing and intensity.
7. Line 389. Prefer higher rather than faster c-intensity.
8. Line 444. "in Figure 1, we see a weaker positive correlation between APHV and PHV, which may be attributed to limitations of the SITAR approach." Please forgive my conflict of interest here, but I don't follow the logic of the statement. What are the SITAR limitations, and how might they generate the positive correlation?
9. Line 445-6. "BMI, … its relationship with the tempo of puberty is less clear than with pubertal timing." Also on lines 447, 450-1 and 457. Please see my original comments about possible confusion between tempo and timing, and I suggest replacing tempo with intensity (it is permissible to use the word intensity without having to precede it with c-).
10. Lines 465-6 state that early puberty is a risk factor for later life decline in bone density, citing my paper [9]. But this is the wrong way round - it is late puberty that is the risk factor.
11. Looking at the GitHub code instructions I note that the SITAR models used age rather than log(age), whereas log(age) has been my recommendation from the outset as it fits consistently better than age and is better justified biologically - see the original SITAR reference [21]. This is a shame, as it would have provided better estimates of b-timing.
12. I previously asked for the numbers of digits in Table 1 to be reduced, and some of them have been. However the PRS P-values remain as two decimal places times a power of ten, which is massively overprecise (even if it may be the convention). There is no need to use any decimal places at all, so nE-p is quite adequate (choose n and p to suit), and even omitting n would lose little. The full model R2 should be consistent, I suggest to 3 decimal places.

Tim Cole

**Reviewer 2**

Bradfield et al. have addressed all of my questions and concerns, or decided some were outside the scope of their study. The manuscript has been greatly improved and I think readers from broad genomics audiences will find it easy to follow. The supplementary note for the analysis plan is well written and quite clear.

**Authors’ response to reviewers**

Abc

**Third round of review**

**Reviewer 1**

abc

**Reviewer 2**

abc

**Authors’ response to reviewers**

abc
